# Mechanisms Underlying Gastrodin Alleviating Vincristine-Induced Peripheral Neuropathic Pain

**DOI:** 10.3389/fphar.2021.744663

**Published:** 2021-12-16

**Authors:** Xiangyu Wang, Boxuan Zhang, Xuedong Li, Xingang Liu, Songsong Wang, Yuan Xie, Jialing Pi, Zhiyuan Yang, Jincan Li, Qingzhong Jia, Yang Zhang

**Affiliations:** ^1^ Departments of Pharmacology, Hebei Medical University, Shijiazhuang, China; ^2^ School of Pharmacy, Hebei Medical University, Shijiazhuang, China; ^3^ Key Laboratory of Innovative Drug Research and Evaluation of Hebei Province, Shijiazhuang, China; ^4^ Laboratory of Neural and Vascular Biology of Ministry of Education, Hebei Medical University, Shijiazhuang, China

**Keywords:** Na_V_1.7/Na_V_1.8, vincristine, neuropathic pain, molecular docking, MD simulation, gastrodin

## Abstract

Gastrodin (GAS) is the main bioactive ingredient of Gastrodia, a famous Chinese herbal medicine widely used as an analgesic, but the underlying analgesic mechanism is still unclear. In this study, we first observed the effects of GAS on the vincristine-induced peripheral neuropathic pain by alleviating the mechanical and thermal hyperalgesia. Further studies showed that GAS could inhibit the current density of Na_V_1.7 and Na_V_1.8 channels and accelerate the inactivation process of Na_V_1.7 and Na_V_1.8 channel, thereby inhibiting the hyperexcitability of neurons. Additionally, GAS could significantly reduce the over-expression of Na_V_1.7 and Na_V_1.8 on DRG neurons from vincristine-treated rats according to the analysis of Western blot and immunofluorescence results. Moreover, based on the molecular docking and molecular dynamic simulation, the binding free energies of the constructed systems were calculated, and the binding sites of GAS on the sodium channels (Na_V_1.7 and Na_V_1.8) were preliminarily determined. This study has shown that modulation of Na_V_1.7 and Na_V_1.8 sodium channels by GAS contributing to the alleviation of vincristine-induced peripheral neuropathic pain, thus expanding the understanding of complex action of GAS as a neuromodulator.

## Introduction

Cancer, characterized by uncontrolled cell proliferation and an absence of cell death, has become the second worldwide cause of death, only exceeded by cardiovascular diseases, seriously threatening the public health (Mattiuzzi and Lippi, 2019). In addition to surgery and radiotherapy, chemotherapy has become the main strategy of cancer treatment (Coffeen et al., 2019), especially the metastatic cancers, and chemotherapeutic drugs are evolving toward increasingly effective treatments. However, chemotherapy-induced neuropathic pain (CINP) is one of the most serious adverse events in the course of chemotherapy (van Schie et al., 2011; Old et al., 2014; Zajaczkowska et al., 2019; Finnerup et al., 2021), and long-term CINP is associated with depression, anxiety, and insomnia, severely impairing the quality of patients’ life and leading to the dose reduction or even cessation of treatment (Rosenthal and Kaufman, 1974; Bär et al., 2005; Jackson et al., 2015; Jha et al., 2021). Currently, the investigation of pathological mechanisms of CINP mainly focuses on the injury of dorsal root ganglion (DRG) sensory neurons, including mitochondrial dysfunction (Argyriou et al., 2013), microfilament and microtubule damage (Sittl et al., 2012), immune-inflammatory response (Zhang and Dougherty, 2014), and ion channels dysfunction (Aromolaran and Goldstein, 2017), and such injury types will lead to ectopic discharges, further contributing to CINP (Doyle and Salvemini, 2021; Yang et al., 2021).

DRG, located between the dorsal horn of the spinal cord and the peripheral nerve terminals, is the cell body of primary afferent neurons, playing a vital role in the transmission and integration of sensory information (Ohshiro et al., 2007; Sapunar et al., 2012). DRG and peripheral axons lack the efficient neurovascular barrier and allow the compounds with larger molecular mass or skeleton to simply diffuse into the interstitium, which is susceptible to the influence of chemotherapeutic drugs and leads to the peripheral pain (Gu and MacDermott, 1997; Kawasaki et al., 2008). In addition, the abnormal expression of TTX-sensitive (Na_V_1.6 and Na_V_1.7) and TTX-resistant (Na_V_1.8 and Na_V_1.9) sodium channels, mainly expressed in DRG neurons, could induce the neuronal hyperexcitability and promoting the development of neuropathic pain (Zimmermann et al., 2007; Ding et al., 2019; Kingwell, 2019; Zhou et al., 2020). Among the above channel proteins, Na_V_1.7 and Na_V_1.8 can be up-regulated under the stimulus of some antitumor agents (such as oxaliplatin and paclitaxel), which is the main cause of CIPN and indicated the vital roles of Na_V_1.7 and Na_V_1.8 in the transmission of pain signals (Argyriou et al., 2013; Zhang and Dougherty, 2014; Vysokov et al., 2019).

Nowadays, the American Society of Clinical Oncology recommends the combination of chemotherapeutics and anti-epileptic (carbamazepine and lamotrigine) or antidepressant drugs (duloxetine and amitriptyline) during chemotherapy to alleviate the severe central side effects (ataxia, conscious confusion), promoting the research and development of higher effectiveness and lower toxicity of analgesic drugs (Hershman et al., 2014; Gül et al., 2020). It’s worth mentioning that there is a famous Chinese herb named Rhizoma Gastrodiae, widely applied as an analgesic. Gastrodin (GAS) is the primary component of Rhizoma Gastrodiae, which is commonly used in the treatment of neurasthenia, vascular headache, pain symptoms caused by radiotherapy or chemotherapy, and neuropathic pain caused by diabetes. GAS has promising physical and chemical properties, especially the polarity and water solubility, and could accumulate in the peripheral nerves and quickly reach the effective drug concentration, showing great potential in the treatment of CINP (Sun et al., 2012; Sun et al., 2016; Xiao et al., 2016; Qin et al., 2021). In this study, a variety of biological and computational experimental methods have been applied to evaluate the efficacy of GAS in the treatment of CINP, and to explore the action mechanisms of GAS alleviating CINP from the molecular level.

## Materials and Methods

### Animals Behavioral Testing

Animal feeding and model establishing methods used in this study complied with the International Association for the Study of Pain Guidelines and have been approved by the Laboratory Animal Ethical and Welfare Committee, the Center for New Drug Safety Evaluation and Research, Hebei Medical University (NO. IACUC-Hebyd AP-2020033). The behavioral measurements on male Sprague Dawley (SD) rats (6–8 weeks) were all done in the awake state. The SD rats weighing 180–220 g were used in this study and were housed individually in automatically controlled environmental conditions, namely 12 h light-dark cycle (lights on from 08:00 to 20:00) and free access to food and water. Prior to the experiment, the rats were placed in the above-mentioned environment for 7 days to fully adapt, and the researchers should observe the rats’ health status every day. SD rats were randomly divided into 3 groups based on mechanical pain threshold (control group, model group, and GAS group) (Qiu et al., 2014; Xiao et al., 2016; Chen et al., 2017; Ye et al., 2018), the specific operations were as follows:

Control group: rats were intraperitoneally injected with the same volume of normal saline (vehicle) daily. Vincristine group: the rats were intraperitoneally injected with vincristine (Shenzhen Wanle Pharmaceutical Co., LTD.) at 9 a.m. every day (DAY 1–7) (0.125 mg/kg, diluted in saline before injection); then normal saline was injected into the abdominal cavity of rats at the same time period (DAY 8–10); finally, vincristine was injected intraperitoneally at the same time (9 a.m.) period on day 11–14. GAS group: DAY 1–7, rats received intraperitoneal injection of vincristine (Shenzhen Wanle Pharmaceutical Co., LTD.) at 9 am daily (0.125 mg/kg, diluted in saline before injection); then rats were injected with normal saline at 9 a.m. and GAS (4-hydroxybenzyl alcohol-4-O--D-glucopyranoside, Purity > 98%, purchased from Nanjing Daosifu Biotechnology Co., Ltd., product batch number: 20170811s) at 16:00 p.m. (60 mg/kg, diluted in saline before injection) from day 8 to 10 daily; finally, rats were intraperitoneally injected with vincristine at 9 a.m. and GAS at 4 p.m. according to the above-mentioned drug dosage (DAY 11–14) (Qiu et al., 2014; Xiao et al., 2016; Chen et al., 2017; Ye et al., 2018).

### Mechanical and Thermal Hyperalgesia

The von Frey instrument was used to assess the threshold sensitivity of mechanical stimuli in rats. The calibrated nylon filaments (von Frey hair, Stoelting) with various bending forces were applied to stimulate the middle plantar surface of the right hind paw of rats. The rats were stimulated from the minimum gram of nylon yarn and the hardness gradually increased. When the hindlimb of the rats was quickly retracted, the rats were considered to have a positive reaction. Hargreaves strategy was applied to measure thermal hyperalgesia using thermal radiation meter (Mengtai Technology). The paw withdrawal latency of the right hind-paw in response to heat was measured using 30% radiant intensity, and the elapsed time was recorded.

### Cell Culture and Electrophysiology

In the DRG neuron excitability recording experiment, the neurons were selected from 32 adult male SD rats (provided by the Experimental Animal Center of Hebei Province, People’s Republic of China), and according to literature reports (Nie et al., 2017; Zhang et al., 2018), DRG neurons were selected from the L4-L6 segment of rats. DRG ganglia were digested with collagenase (3 mg/mL) and dispase (7.5 mg/mL) for 30 min at 37°C, and then mechanically triturated and washed twice with DMEM supplemented with 10% fetal calf serum, which were further plated on poly-D-lysine-coated glass coverslips. Next, the action potential, Na_V_1.8 current density, and TTX-sensitive sodium current were recorded.

#### Action Potential Recording

In this part, the selected DRG neurons were derived from the constructed rat model of vincristine and treated with different concentrations of GAS to observe the effects of GAS on the excitability of model neurons. Based on previous literature reports, GAS bath solutions with concentrations of 30, 100, and 200 µM were configured in reference to the effective GAS concentration (about 90 µM) (Qiu et al., 2014; Nepal et al., 2019; Qi et al., 2019). The configured GAS bath solution directly perfused the DRG model neurons for 10 min to observe the effect of GAS on the excitability of the neurons.

Action potentials of dissociated rat small-diameter DRG neurons (17–25 μm) were recorded with a current clamp using the HEKA EPC10. Pipettes (1–6 MΩ) were filled with the solution containing the following components: KCl (140 mM), CaCl_2_ (1 mM), MgCl_2_ (2 mM), HEPES (10 mM), EGTA (11 mM), and the pH of the solution was adjusted to 7.4 with NaOH. Small DRG neurons were injected with various currents with different intensities (ranging from 0 to 500 pA with 10 pA as the gradient) to examine the action potential. This experiment mainly detects the action potential amplitude, threshold, rheobase, depolarization slope (V/s), etc.

#### Na_V_1.8 Sodium Currents Recording

GAS was dissolved in bath solution containing specific components, and then used to treat DRG neurons from vincristine model rats for 10 min to observe the effects of GAS on Na_V_1.8 channel current. The specific operations were as follows:

The Na_V_1.8 current of small-diameter model DRG neurons was recorded in whole-cell configuration by voltage-clamp. The pipettes solution mainly consisted of CsCl (70 mM), NaCl (30 mM), TEA-cl (30 mM), EGTA (10 mM), CaCl_2_ (1 mM), MgCl_2_ (2 mM), HEPES (10 mM), D-glucose (5 mM), Na_2_ATP (2 mM), GTP (0.05 mM), and the pH value was adjusted to 7.3 by CsOH. The bath solution (pH = 7.4) contained NaCl (80 mM), Choline-Cl (50 mM), TEA-Cl (30 mM), CaCl_2_ (2 mM), CdCl_2_ (0.2 mM), HEPES (10 mM), and D-glucose (5 mM). Since Na_V_1.8 and Na_V_1.9 were TTX-resistant channels in contrast to TTX-sensitive Na_V_1.7, Tetrodotoxin (TTX, 500 nM) was added to block its sensitive sodium channel currents and retain TTX-resistant Na_V_1.8 and Na_V_1.9 sodium channels. Compared with Na_V_1.8, Na_V_1.9 was inactivated at a relatively high voltage. Therefore, when the prepulse voltage was set to −44 mV, 500 ms, the Na_V_1.9 current was inactivated and the Na_V_1.8 current was separated. The specific operations were as follows: the preset voltage of −44 mV, 500 ms was set to inactivate the Na_V_1.9 current, and then the Na_V_1.8 current was excited using a series of 50 ms steps depolarization (−80–0 mV in 5 mV increments) (Cummins et al., 1999; Tyrrell et al., 2001). The HEKA EPC10 has an acquisition rate of 20 kHz, and the signals were filtered at 5 kHz.

#### Na_V_1.7 Sodium Currents Recording

The stable expression system of Na_V_1.7 on HEK239B cell line was provided by Inovogen (Inovogen Tech. Co., Beijing, China), and the construction method was roughly divided into the following four steps: 1) The full-length Na_V_1.7 gene (SCN9A) was obtained using gene synthesis method and constructed into the transposon vector (pTP6-puro) by KpnI-NotII. 2) The plasmid was extracted with bacterial solution containing pTP6-puro-1.7 and transfected into HEK239B cells, followed by polyclonal screening with 3 µg/ml puromycin. After 2 weeks of screening, the culture medium was changed or puromycin was added again after passages. 3) The selected polyclonal cells were frozen until no new dead cells appeared in the clone system. 4) Finally, Realtime PCR was used to detect the expression of target genes. Based on the above method, the vitro expression system of Na_V_1.7 was constructed to directly record the current changes of Na_V_1.7. In addition, the primary structure of the expression system was determined by Inovogen, which was aligned to the sequence template from *Rattus norvegicus* (NP_579823.1) using blastx (99% identity).

The Na_V_ 1.7 current of HEK293B was recorded in whole-cell configuration by voltage-clamp. The pipettes solution contained CsF (145 mM), NaF (5.6 mM), HEPES (5 mM), and the pH was adjusted to 7.3 using CsOH. The acquisition rate was 20 kHz, and signals were filtered at 5 kHz. The protocol was set to increase from −80 to 20 mV (increments of 10 mV each time) to evoke the Na_V_1.7 current, and each stimulation last for 50 ms. The applied bath solution contained NaCl (140 mM), KCl (5.4 mM), CaCl_2_ (1.8 mM), and MgCl_2_ (0.5 mM), HEPES (5 mM), D-glucose (5.5 mM), NaH_2_PO_4_ (0.4 mM), and the pH was adjusted to 7.4 with NaOH.

#### TTX-Sensitive Sodium Current of DRG Neurons Recording

The TTX-sensitive sodium current of small-diameter model DRG neurons was recorded in whole-cell configuration by voltage-clamp. The pipettes solution mainly consisted of CsCl (70 mM), NaCl (30 mM), TEA-cl (30 mM), EGTA (10 mM), CaCl_2_ (1 mM), MgCl_2_ (2 mM), HEPES (10 mM), D-glucose (5 mM), Na_2_ATP (2 mM), GTP (0.05 mM), and the pH value was adjusted to 7.3 by CsOH. The bath solution (pH = 7.4) containing NaCl (80 mM), Choline-Cl (50 mM), TEA-Cl (30 mM), CaCl_2_ (2 mM), CdCl_2_ (0.2 mM), HEPES (10 mM), and D-glucose (5 mM). Total sodium current was elicited by the stimulation of −10 mV, 50 ms, and the TTX-sensitive sodium current was obtained by subtracting the current after TTX processing from the total Na current.

### Quantitative PCR

According to the instructions, 800 ng RNA was reversely transcribed using PrimeScriptTM RT reagent Kit with gDNA Eraser (perfect real time) kit (Takara, Japan) (Wang et al., 2017; Wang et al., 2021); then, gene-specific mRNA analyses were conducted with SYBR premix ex TaqTMⅡ (TliRnaseH plus) kit (Takara, Japan) as standard protocol. In this study, Gapdh was applied as a reference to normalize the mRNA expression of SCN9A and SCN10A. After amplification, each qPCR product was electrophoresed to ensure specificity. After the components required for the PCR reaction were configured, the cycling systems were placed on the PCR machine and preheated at 95°C for 3 min to fully denature the template DNA, and then enter the amplification cycle stage.

In each cycle, the template was denatured at 95°C for 30 s, and then the temperature was lowered to the renaturation environment of 60°C for 30 s to fully anneal the primer and template. Finally, the prepared systems were kept in 72°C for 1 min (amplifying 1 kb fragment) to make the primer extend on the template and synthesize DNA. The above cycle was repeated 39 times to accumulate a large amount of amplified DNA fragments, and then kept at 72°C for 5 min to complete the extension of the products. Finally, the products were preserved at 4°C.

The genbank accession number of SCN9A and SCN10A were NM_133289.1 and NM_017247.1, respectively. The position of primer sequences of SCN9A located between 1174 and 1293 base pairs, and the primer sequences of SCN10A were located between 581 and 737 base pairs. In addition, SCN9A was the gene expressing the Na_V_1.7 channel, and the primer was detailed in the following sequence: Na_V_1.7 (SCN9A): forward TAC​CTG​ATA​AAC​TTG​ATC​CTG​GC; reverse TTT​GAG​TCG​GTC​TAA​CAT​CTG​CT; SCN10A was the gene expressing the Na_V_1.8 channel, and the primer was detailed in the following sequence: Na_V_1.8 (SCN10A): forward GTC​TGT​CCA​TTC​CTG​GTT​CTC​C; reverse ACA​AAA​CCC​TCT​TGC​CAG​TAT​CT.

### Western Blot

The DRG neuron lysates were prepared with RIPA lysis buffer, and the protein loading amount was 40 µg. Equal amounts of protein were separated by SDS-PAGE and electro-transferred to the polyvinylidene fluoride (PVDF) membrane. PVDF membrane was blocked with 5% nonfat dairy milk, and incubated with primary antibody of Na_V_1.7, Na_V_1.8, and β-actin overnight at 4°C, which was further incubated with IRDye800-conjugated secondary antibody (1: 50,000; EARTHOX) for 2 h at room temperature and subsequently scanned with the Odyssey Infrared Imaging System (LI-COR). The primary antibody of Na_V_1.7 (item number ARG56140) was monoclonal antibody provided by Shanghai Bio-Platform Technology Company (Shanghai, China), and the host was mice. The production number of Na_V_1.8 was ARG56141, and the rest of the information was consistent with Na_V_1.7. Monoclonal antibody for mouse β-actin (product number 66009-1-Ig) was provided by proteintech (Rosemont, IL, United States). The integrated intensity of polyvinylidene fluoride membrane was detected by Odyssey Imager software (version 3.0).

### Cell Immunofluorescence Pretreatment and Structured Illumination Microscopy Image Preparations

The model group neurons were incubated with vincristine for 24 h, and the GAS group was incubated with the mixture of GAS and vincristine for 24 h. DRG neurons were collected after washed by PBS for 3 times, and then washed with 4% paraformaldehyde for 30 min, which were transferred to 3% BSA and 0.3% Triton solution for 60 min. DRG neurons were blocked with 10% normal goat serum containing 0.3% Triton-X-100 (co-incubation for 1 h, 37°C). The sections were incubated with primary antibody for 12 h at 4°C (anti-Nav1.7 channel antibody: ARG56140; anti-Nav1.8 channel antibody: ARG56141), which were washed 5 times with PBS solution for minutes each time. The sections were incubated with secondary antibody for 2 h at room temperature, and then the secondary antibody was removed using PBS solution. Finally, the sections containing DRG were sealed with gelatin coating and placed in the dark for SIM imaging.

### DRG Tissue Immunofluorescence Preparation

The ascending aorta of SD rats was perfused with saline solution, and then perfused with 4% paraformaldehyde (PFA, pH 7.4, 4°C). Subsequently, the DRG L5 ganglion was removed and fixed in 4% PFA for 24 h (4°C), and then soaked in 30% sucrose for 48 h (4°C) to dehydrate. After that, the dehydrated ganglion was sectioned into 14 μm thick slices in the cryostat using gelatinized slides, and then subjected to immunofluorescence treatment. Then, the sections were blocked with 0.2% Triton X-100 containing 2% BSA for 1 h at room temperature, and then incubated with primary antibody (anti-Na_V_1.7 channel antibody: ARG56140; anti-Na_V_1.8 channel antibody: ARG56141) overnight at 4°C. Finally, the sections were washed 3 times with PBS at room temperature (5 min for each time), and then co-incubated with secondary antibodies at room temperature for 1 h.

### Molecular Docking

We constructed the three-dimensional structures of Na_V_1.7&1.8 using homology modeling method.

In this study, Discovery Studio 2020 Client (DS 2020) was used for the prediction of binding pockets, molecular docking, and the estimation of the binding interactions between the receptor and GAS. Molecular docking was performed by CDOCKER module with the CHARMm force field. First, the three-dimensional (3D) structures of Na_V_1.7 and Na_V_1.8 were constructed using homology modeling method (Waterhouse et al., 2018), and processed by “prepare proteins module,” including Loop domain optimization, protonation, removing water, energy minimization using conjugate gradient algorithm with CHARMm force field. Second, GAS was sketched by ChemDraw 19.0, and prepared by the “Prepare Ligands module,” consisting of pH-based ionization, tautomers generation, generating 3D coordinates, rearranging hydrogens, and minimization. Then, the binding sites of the receptor were defined by the “From Receptor Cavities” method within the “Define and Edit Binding Site module,” and referring to the active sites reported in the literature (Ito et al., 1989; Swain et al., 2017; Moyer et al., 2018; Wu et al., 2018; Xu et al., 2019). Finally, the CDOCKER method was applied to evaluate the binding interactions of GAS at different sites with the docking score as an important criterion (Zhang et al., 2019a; Zhang et al., 2020a).

### Molecular Dynamic Simulation

Molecular dynamic (MD) simulation was further used to evaluate the binding interactions between the GAS and the predicted pockets. MD simulations of the constructed systems were performed using GPU-accelerated PMEMD in Amber18 on 28 cores of an array of two 2.9 GHz Intel Gold 6226R processors and 6 pieces of Nvidia Tesla v100s 32 GB graphic card. Prior to MD simulation, all components (protein-ligand complex, protein, and ligand) were processed with program tleap embedded in AmberTools to generate the corresponding coordinate files and topology files. Amber ff14SB and general Amber force fields were applied for the receptor protein and the docked ligand, and the Li/Merz ion parameters for SPC/E water model were directly used based on the previous publications (Wang et al., 2018; Wang et al., 2019; Zhang et al., 2020b; Li et al., 2021; Liang et al., 2021). Antechamber was chosen to assign the charges of the docked ligands via the restrained electrostatic potential partial charges, and Gaussian 09 was used to optimize the ligand’s geometry and calculate the electrostatic potential calculations at HF/6–31G* level. Then, the processed systems were subjected to 200 ns MD simulation, mainly consisting of 2 steps energy minimization optimization process, progressive heating process, 5 ns equilibrium process, and the final 200 ns MD process. Program cpptraj was used to analyze the RMSD and representative conformations of MD trajectories, and mm_pbsa.pl program was selected to explore the binding free energies between the ligand and receptor.

### Statistical Analysis

The behavioral experiment results were processed and analyzed by OriginPro (version 9.1.0). Statistical difference among multiple groups was compared by one/two-way ANOVA, and Bonferroni Test was used for two group comparisons, which was presented as mean ± SD. HEKA FitMaster (version 2x90.3) and OriginPro (version 9.1.0) were used to analyze the electrophysiological data, and the above-mentioned method was applied to analyze and compare the statistical difference, presented by mean ± SEM. Threshold represents the voltage value at the inflection point during the rising phase of the action potential, recorded with the current clamp (Zhang et al., 2019b). Additionally, $G=I/\left(V_{m}-E_{Na}\right)$ was applied to convert the peak inward currents obtained from activation protocols (*G* represented the conductance; *I* indicated the peak inward current; $V_{m}$ represented the instantaneous membrane potential; $E_{Na}$ indicated the equilibrium potential for sodium channel.) Maximum conductance value was used to normalize the conductance data, which was further fit with Boltzmann equation, namely $G=G_{max}+\left(G_{min}-G_{max}\right)/\left(1+\text{exp}\left[\left(V_{m}-V_{1/2}\right)/k\right]\right)$ (*G* also represented the conductance; $V_{1/2}$ was the midpoint of activation; *k* indicated the slope factor.). The IC_50_ values were calculated using logistic equation fitting. Licor Odyssey software was applied to quantify the gray values of proteins in WB, and fluorescence intensity in immunofluorescence was measured by NIS-Elements Viewer (version 4.20, Nikon, Japan) software.

## Results and Discussion

### Alleviation of GAS on Vincristine-Induced Mechanical and Thermal Hyperalgesia in Rat

**FIGURE 1:**
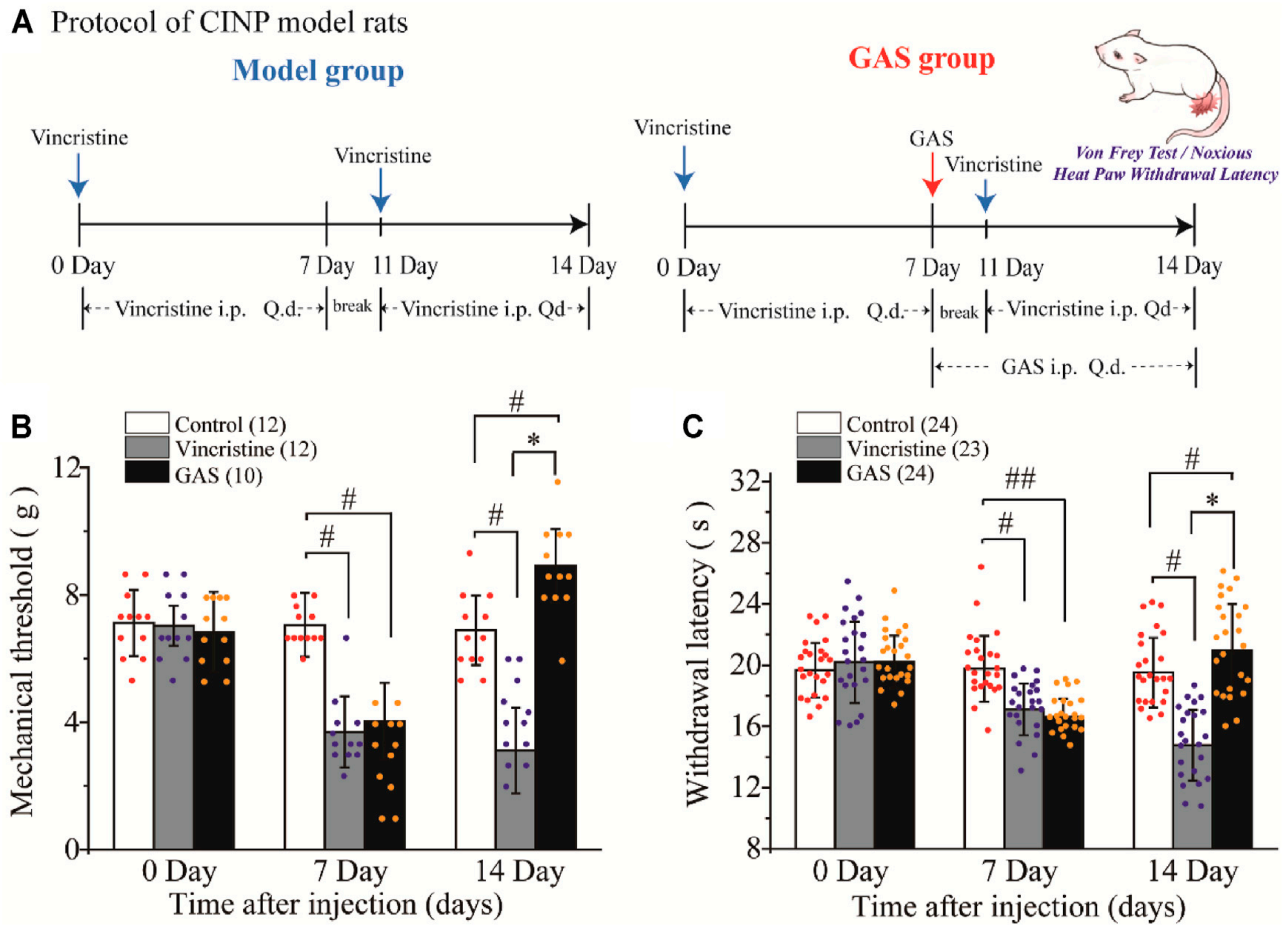
The effects of GAS on alleviating the symptoms of vincristine-induced CINP rats: **(A)** Administration protocol of GAS in the treatment of neuropathic pain model rats caused by vincristine; **(B)** The effects of GAS against vincristine-induced hyperalgesia on mechanical paw withdrawal duration in a pinprick test, and the mechanical paw withdrawal threshold was measured at 0, 7, 14 Day; **(C)** Effects of GAS on plantar thermal pain in vincristine model rats, and the thresholds were measured at 0, 7, 14 Day (*#p* < 0.05, *##p* < 0.01, compared to the control group; *p < 0.05, compared to the model group; *ANVOA- Bonferroni Test*).

Vincristine has been reported to induce various pains, such as postherpetic neuralgia, diabetic neuralgia (Feld et al., 1980), and type I complex regional pain syndrome (Benzon et al., 2003), which has been associated with damage to the peripheral nerve terminal (Rosenthal and Kaufman, 1974; Dupuis et al., 1985; Old et al., 2014). Additionally, more than half of the rats treated with vincristine have been found to respond abnormally to thermal and mechanical pain induced by C fiber stimulus (Tanner et al., 1998; Xiao and Bennett, 2008; Xu et al., 2017). Therefore, vincristine was used to construct the CINP rat model, and thresholds of mechanical and thermal pains were measured (Figure 1A).

There was no difference in the mean paw withdrawal threshold of the three groups as determined by von Frey and radiant heat stimulus. The selected experimental rats were randomly divided into three groups (control group, vincristine group, and GAS group), and the basic values of mechanical and thermal thresholds were preliminarily evaluated before drug administration. The specific values were as follows: mechanical thresholds: 7.11 ± 1.03 g (control group), 7.02 ± 0.62 g (vincristine group), 6.83 ± 1.25 g (GAS group); thermal thresholds: 19.65 ± 1.78 s (control group), 20.19 ± 2.65 s (vincristine group), 20.21 ± 1.73 s (GAS group). After 7 days of vincristine administration (ip, 0.125 mg/kg), the values of mechanical and thermal thresholds were calculated, and the mean paw withdrawal threshold was significantly reduced compared with the control group: mechanical thresholds: 7.05 ± 1.00 g (control group), 3.69 ± 1.11 g (vincristine group), 4.02 ± 1.21 g (GAS group); thermal thresholds: 19.76 ± 2.15 s (control group), 17.11 ± 1.68 s (vincristine group), 16.63 ± 1.16 s (GAS group).

Continuous administration of GAS (ig, 60 mg/kg) for 7 days significantly attenuated the development of mechanical (*n* = 10) and thermal hyperalgesia (*n* = 23), as shown in Figure 1B. Quantitative analysis showed that, compared with the vincristine model, GAS increased the mechanical pain threshold from 3.11 ± 1.34 g to 8.92 ± 1.14 g, and the thermal withdrawal latency from 14.77 ± 2.32 s to 20.97 ± 3.01 s on the 14th day of modeling (mean ± SD, ***p < 0.001 and ***p < 0.001) (Figure 1C). Apparently, the applied modeling method successfully induced mechanical pain and thermal hyperalgesia in rats. After treatment with GAS, the thresholds of mechanical and thermal hypersensitivity were greatly improved, and the mechanical tactile and thermal allergic of the model rats could be restored to normal levels. In addition, in order to better illustrate the mechanisms underlying GAS alleviating vincristine induced peripheral neuropathic pain, the influences of GAS on the mechanical and thermal thresholds in normal SD rats were also observed. The results showed that the mechanical and thermal thresholds didn’t increase significantly after 3 days of GAS administration, indicating that the mechanism of GAS reversing vincristine-induced hyperalgesia was different from that of anesthetic effects (Supplementary Figure S1). The behavioral results suggested that GAS had a good curative effect on CINP model rats, which was expected to be a small active molecule for the treatment of peripheral neuropathic pain.

### Inhibitory Effects of GAS on the Hyperexcitability of Small-Sized DRG Neurons Induced by Vincristine

**FIGURE 2:**
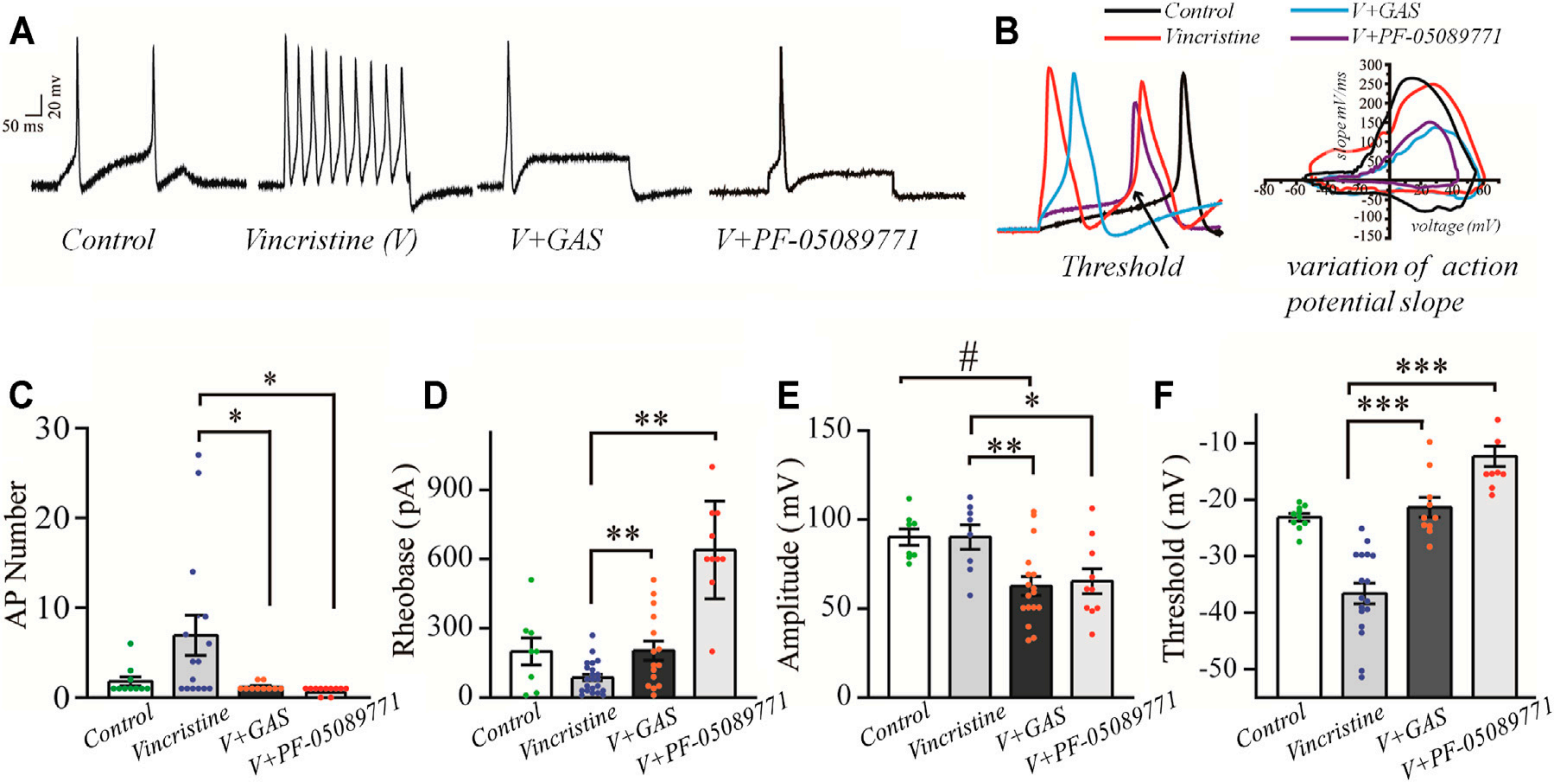
The influences of GAS on the excitability of vincristine-induced hyperexcitability of small-sized DRG neurons: **(A)** typical action potential curve of spike firing of each experimental group; **(B)** phase plot of the action potential **(left),** and the raising slope of action potential **(right);** **(C)** the effect of GAS on the number of action potentials fired by DRG neurons in each group; **(D)** histogram of the effects of GAS on action potential rheobase of DRG neurons in each group; **(E)** the influence of GAS on the amplitude of the first peak of action potential fired by DRG neurons; **(F)** regulation of GAS on the firing threshold of action potentials of DRG neurons in each group (*#p* < 0.05, ###*p* < 0.001, compared to the control group; *p < 0.05, **p < 0.01, ***p < 0.001, compared to the model group; ANVOA- Bonferroni Test).

As primary sensory afferent neurons, the ectopic afferent discharge of DRG neurons is widely considered to be the main cause of chronic pain after peripheral nerve injury (Seijffers et al., 2007; Norcini et al., 2016). Thus, DRG neurons were isolated to observe the changes of their firing patterns. The alleviation of vincristine-induced rat hyperalgesia by GAS indicated that GAS might directly decrease the excitability of neurons. In order to explore the mechanism of GAS, the firing spikes of action potentials were recorded on small-diameter (17–25 μm) DRG neurons, which were closely related to the afferent noxious signal. GAS (100 μM) significantly attenuated hyperexcitability of vincristine-induced DRG neurons, and reduced the number of action potentials triggered by 500 pA (Figures 2A,C). 200 μM GAS and 500 nM PF-05089771 (Cat. No.: HY-12883, MCE) notably increased the rheobase current (depolarization current threshold eliciting the 1st action potential), namely, from 86.8 ± 3.1 pA (*n* = 22) to 203.3 ± 10.7 pA (*n* = 15, **p < 0.01) and 640.0 ± 66.2 pA (*n* = 10, **p < 0.01) (Figure 2D); the action potential threshold voltage also remarkably increased from −36.6 ± 0.7 mV (*n* = 17) to −21.3 ± 1.8 mV (*n* = 10, ***p < 0.001) and −12.3 ± 1.8 mV (*n* = 10, ***p < 0.001) (Figure 2F); 200 μM GAS and 500 nM PF-05089771 significantly reduced the amplitude of action potentials in DRG neurons (**p < 0.01) (Figure 2E). Figure 2B showed that GAS decreased the rate of action potential slope rise (dV/dt). Therefore, GAS could significantly reduce the number of action potential bursts of DRG neurons in model rats, decrease the amplitude of action potentials, and increase the rheobase and threshold of action potentials (Table 1), thereby inhibiting the excitability of DRG neurons in model rats, which was similar to that of the inhibitory effects of PF-05089771 on excitability of model DRG neurons.

**TABLE 1:** Summarized effects of GAS on vincristine-induced hyperexcitability of the action potential in DRG neuron.

Groups	RMP (mV)	Rheobase (pA)	Amplitude (mV)	AP duration (ms)	AP number
Control	−49.6 ± 0.9 (10)	200.0 ± 16.5 (8)	90.2 ± 1.6 (8)	13.9 ± 0.7 (10)	1.8 ± 0.2 (10)
Vincristine	−45.53 ± 0.8 (10)	86.8 ± 3.1 (22)^c^	90.1 ± 2.4 (8)	6.5 ± 0.3 (10)^d^	6.9 ± 0.6 (15)
V+GAS 30 μM	−47.1 ± 0.4 (12)	108.0 ± 6.8 (10)	80.4 ± 2.8 (8)	6.7 ± 0.7 (10)^d^	4.3 ± 0.6 (10)
V+GAS 100 μM	−48.5 ± 0.9 (11)	120.9 ± 7.2 (11)	71.7 ± 2.3 (8)^c^	7.8 ± 0.3 (10)^c^	3.6 ± 0.6 (10)
V+GAS 200 μM	−47.2 ± 0.5 (15)	203.3 ± 10.7 (15)^b^	62.6 ± 1.3 (17)^b^ ^,^ ^d^	9.0 ± 0.9 (10)	1.2 ± 0.04 (10)^a^

a Compared with vincristine group, *p* < 0.05.

b Compared with vincristine group, *p* < 0.01.

c Compared with control group, *p* < 0.05.

d Compared with control group, *p* < 0.01. All data are given as the mean ± SEM.

The repetitive firing of DRG neurons depends on the inherent resonance characteristics of the cell membrane. Most small or medium-diameter DRG neurons cannot produce repeated firing during continuous depolarization, and step depolarization stimulus only causes a single spike or transient burst (Amir and Devor, 1996; Amir et al., 2005). According to Figures 2A,C, it could be found that vincristine significantly increased the number of repetitive discharges of DRG neurons, and the increase of ectopic discharges was the internal cause of neuropathy-induced pain sensation. GAS effectively suppressed the number and amplitude of action potential, thereby reducing the occurrence of hyperalgesia. In addition, GAS also significantly increased the thresholds and rheobase of action potentials and reduced the probability of action potential bursts. Furthermore, GAS reduced the upstroke slope of action potential’s rising phase, and slowed down the depolarization of the action potential, thus retarding the occurrence process of the action potential. Furthermore, based on the effects of GAS on the excitability of DRG neurons in normal SD rats, it could be found that GAS could increase the threshold and rheobase of action potentials to a certain extent, but had fewer effects on the number and amplitude of action potential, indicating that DRG neurons from normal SD rats were less sensitive to GAS compared to that of model DRG neurons (Supplementary Figure S2). In short, based on the biological functions of GAS discussed above, GAS reduced vincristine-induced hyperalgesia of primary sensory neurons associated with pain.

### Inhibitory Activities of GAS Against Na_V_1.7 and Na_V_1.8 Sodium Channel Currents From the Cells Pre-Treatment With Vincristine

#### The Influence of GAS on the Kinetic Process of Na_V_1.7 Channel

Na_V_1.7 has been recognized as an important target in the nociceptive pathway, and the peripheral expression of the Na_V_1.7 channel can stimulate pain signals in the DRG neurons by promoting minor stimulation, prompting the release of neurotransmitters at the first synaptic site in the spinal cord and participating in pain signal transduction (Kingwell, 2019). Na_V_1.7 is expressed on the sensory neurons or nociceptors, depolarizes the cell membrane of the injured site by mediating the inward flow of sodium ions, thereby triggering the firing of action potentials and mediating the transmission of pain signals (Waxman and Zamponi, 2014; Xia et al., 2016; Kingwell, 2019).

**FIGURE 3:**
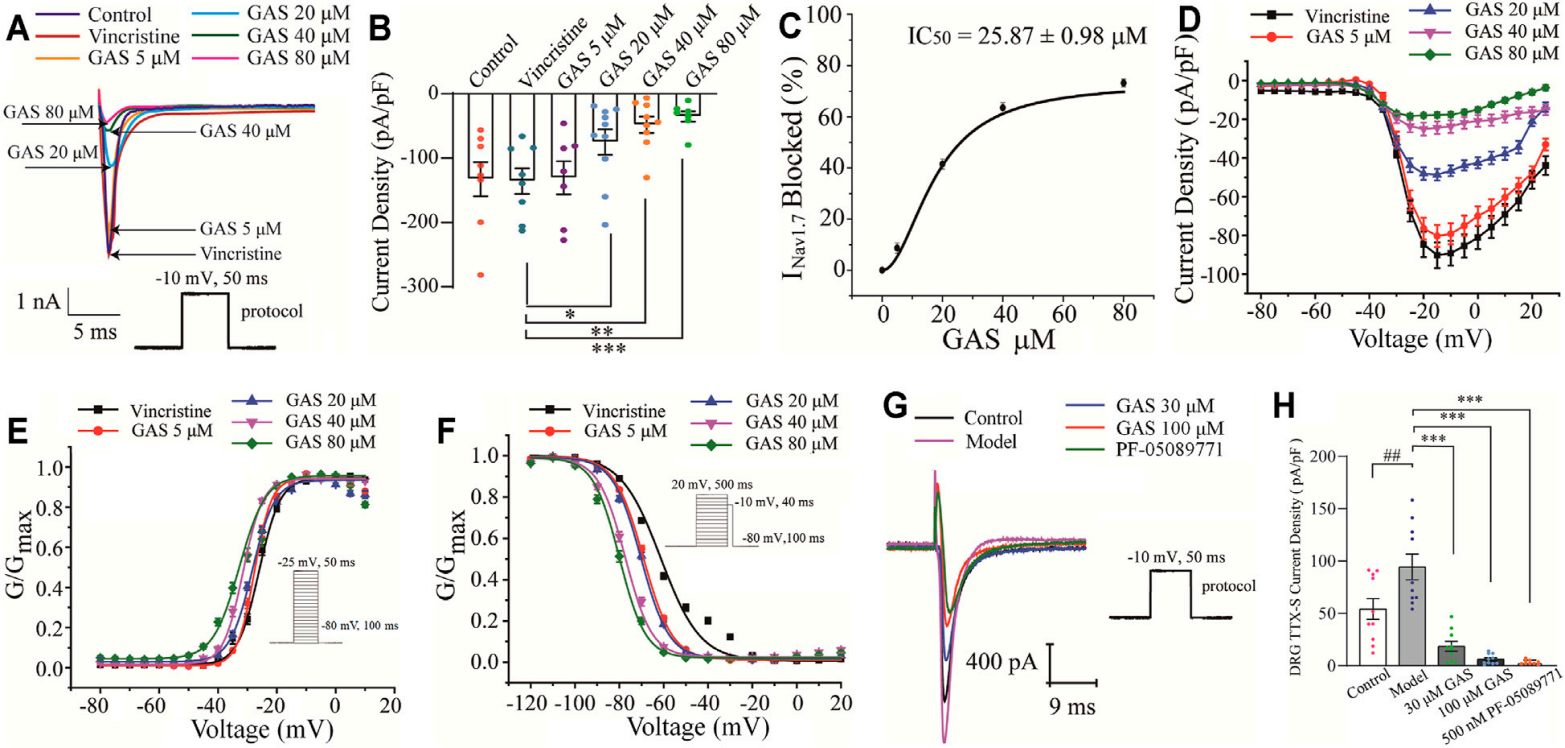
Influences of GAS on Na_V_1.7 channel current: **(A)** typical inhibition curve of GAS on Na_V_1.7 channel current; **(B)** histogram of the influence of GAS on Na_V_1.7 channel current density; **(C)** concentration-response curves of GAS on Na_V_1.7 current (IC_50_ = 25.87 ± 0.98 μM); **(D)** the influence of different concentrations of GAS on the I-V curve of Na_V_1.7 channel current density; **(E)** the influence of GAS on the activation curve of Na_V_1.7 channel; **(F)** the voltage-dependent inactivation curves of G_Nav1.7_; **(G)** the typical curve of GAS inhibiting the sodium current of DRG neurons; **(H)** the effects of GAS on the TTX-sensitive sodium current of DRG neurons (##*p* < 0.001, compared to the control group; *p < 0.05, **p < 0.01, ***p < 0.001, compared to the model group; ANVOA- Bonferroni Test).

In this part, the whole-cell patch-clamp was applied to examine the effects of GAS on the over-expressed Na_V_1.7 channel current on the HEK239B cell line. Firstly, the selected cell line was pre-incubated with vincristine (30 µg/L) for 24 h, and then the Na_V_1.7 channel currents were recorded with different concentrations of GAS (5, 20, 40, 80 μM), making the current density decrease significantly from −135.7 ± 7.0 pA/pF (*n* = 10) to −30.7 ± 9.8 pA/pF (*n* = 10), −74.9 ± 6.2 pA/pF (*n* = 8), *p < 0.05), −47.8 ± 4.2 pA/pF (*n* = 8, **p < 0.01), −35.1 ± 3.1 pA/pF (*n* = 6, ***p < 0.001) (Figures 3A,B). Obviously, GAS presented a dose-dependent inhibition of the current density of Na_V_1.7. In addition, Hill fitting was used to calculate the IC_50_ value of GAS’s inhibitory activities against Na_V_1.7 (IC_50_ = 25.87 ± 0.98 μM), as shown in Figures 3C,D.

Voltage-dependent activation and steady-state inactivation are vital characteristics of ion channels directly influencing the excitability of cells. The Na_V_1.7 channel is characterized by rapid activation, rapid inactivation, and slow resurgent (Herzog et al., 2003). Compared with other sodium channels, the activation and inactivation curves of Na_V_1.7 current present a hyperpolarization trend, generating a larger inward current and then activating the Na_V_1.8 channel on the nociceptive receptors. Thus, the Na_V_1.7 current activation experiment and the Na_V_1.7 current inactivation experiment were performed using a patch clamp. The current density-voltage relationship was converted to the conductance (G_Nav1.7_)-voltage, which was fitted into the Boltzmann equation. Compared with the vincristine group, the activation curve and steady-state inactivation curve of the Na_V_1.7 channel in the GAS group shifted to the hyperpolarization direction, and the effects of GAS on the steady-state inactivation was more obvious. The slopes of the activation and inactivation curves of the Na_V_1.7 channel remained basically unchanged (Figures 3E,F), and the half-maximum activation voltage (V_1/2_) of G_Nav1.7_ changed a little (Figure 3E; Table 2). However, the inactivated V_1/2_ of G_Nav1.7_ shifted to the hyperpolarized direction, and the movement effect was observed in a dose-dependent manner (Figure 3F; Table 2). In summary, GAS mainly suppressed the inward current of the Na_V_1.7 channel and promoted the inactivation state of the Na_V_1.7 channel.

**TABLE 2:** The effect of GAS on V_1/2_ of Na_V_1.7 channel and Na_V_1.8 channel in cells.

	Na_V_1.7 channel V_1/2_			Na_V_1.8 channel V_1/2_	
	Activation	Inactivation		Activation	Inactivation
Vincristine	−26.1 ± 0.3	−61.3 ± 1.6	Control	−24.1 ± 1.2	−35.2 ± 1.4
GAS 5 μM	−27.6 ± 0.7	−69.1 ± 0.8	Model	−33.7 ± 0.7	−24.2 ± 1.2
GAS 20 μM	−28.4 ± 0.5	−70.2 ± 1.2	GAS 3 μM	−32.6 ± 1.3	−27.8 ± 1.3
GAS 40 μM	−32.3 ± 0.7	−77.3 ± 1.1	GAS 10 μM	−34.4 ± 0.8	−29.1 ± 1.2
GAS 80 μM	−32.7 ± 1.2	−79.9 ± 1.2	GAS 30 μM	−29.2 ± 1.1	−37.4 ± 1.9
			GAS 100 μM	−34.4 ± 0.6	−34.4 ± 1.0
			GAS 300 μM	−31.9 ± 1.1	−37.7 ± 1.9
			GAS 1,000 μM	−32.7 ± 1.4	−37.3 ± 0.3

According to the above results, GAS produced a large slope current by directly inhibiting the Na_V_1.7 channel current, making the action potential produce small and slow depolarization in the process of discharge, and thus playing the regulatory role of reducing neuronal excitation. GAS mainly promoted the inactivation of the Na_V_1.7 channel, making the channel more likely to be inactivated or more sodium channels be in an inactive state under a negative membrane potential (Figures 3E,F). Similar to other small sodium channel blockers, most of the blockers currently being developed around Na_V_1.7 make the channels be in the fast or slow inactivation state, or prolonging the process of resurrection, reducing the number of sodium channels contributing to the action potential and inhibiting the abnormal firing of action potentials.

Besides, the effects of GAS on TTX-sensitive sodium current were recorded on DRG neurons. While Na_V_1.6 was mainly distributed in medium and large diameter neurons (Chen et al., 2020), the small-diameter neurons that were concerned in this study expressed Na_V_1.7 (TTX-sensitive), Na_V_1.8 and Na_V_1.9 (TTX-resistant) (Wang et al., 2020). In this section, since the current of Na_V_1.7 couldn’t be directly observed, the TTX-sensitive sodium current obtained by subtracting the current after TTX processing from the total Na current, indicating the large component of the TTX-sensitive sodium current was Na_V_1.7. Furthermore, according to Figures 3G,H, it could be found that TTX-sensitive sodium current density of model DRG neurons decreased from −94.3 ± 12.4 pA (*n* = 10) to −18.4 ± 4.7 pA (*n* = 10), −18.4 ± 4.7 pA (*n* = 10) to −6.1 ± 1.6 pA (*n* = 9) after being treated with 30 μM, 100 μM GAS, respectively. Moreover, such TTX-sensitive sodium current density couldn’t be further inhibited by selective Na_V_1.7 inhibitor PF-05089771 [from −6.1 ± 1.6 pA (*n* = 9) to −3.3 ± 0.6 pA (*n* = 10)]. The experimental results showed that the above TTX-sensitive sodium channels were very sensitive to low concentration of GAS (30 μM), and most of the current could be inhibited by GAS, consisting with the results recorded in HEK293B cell line.

#### The Influence of GAS on the Na_V_1.8 Channel

Since the Na_V_1.8 channel plays a vital role in the rising phase of the action potential, it has a profound influence on the evoked discharge of the action potential (Waxman and Zamponi, 2014; Xia et al., 2016; Klein et al., 2017). The depolarization activation and the slow inactivation of Na_V_1.8 are related to the repetitive firing of DRG neurons. It was observed in Figures 2C,E that GAS could significantly decrease the action potential amplitude and reduce repetitive discharges of CINP model rats. Therefore, it is necessary to further explore the influence of GAS on the current and inactivation of the Na_V_1.8 channel.

**FIGURE 4:**
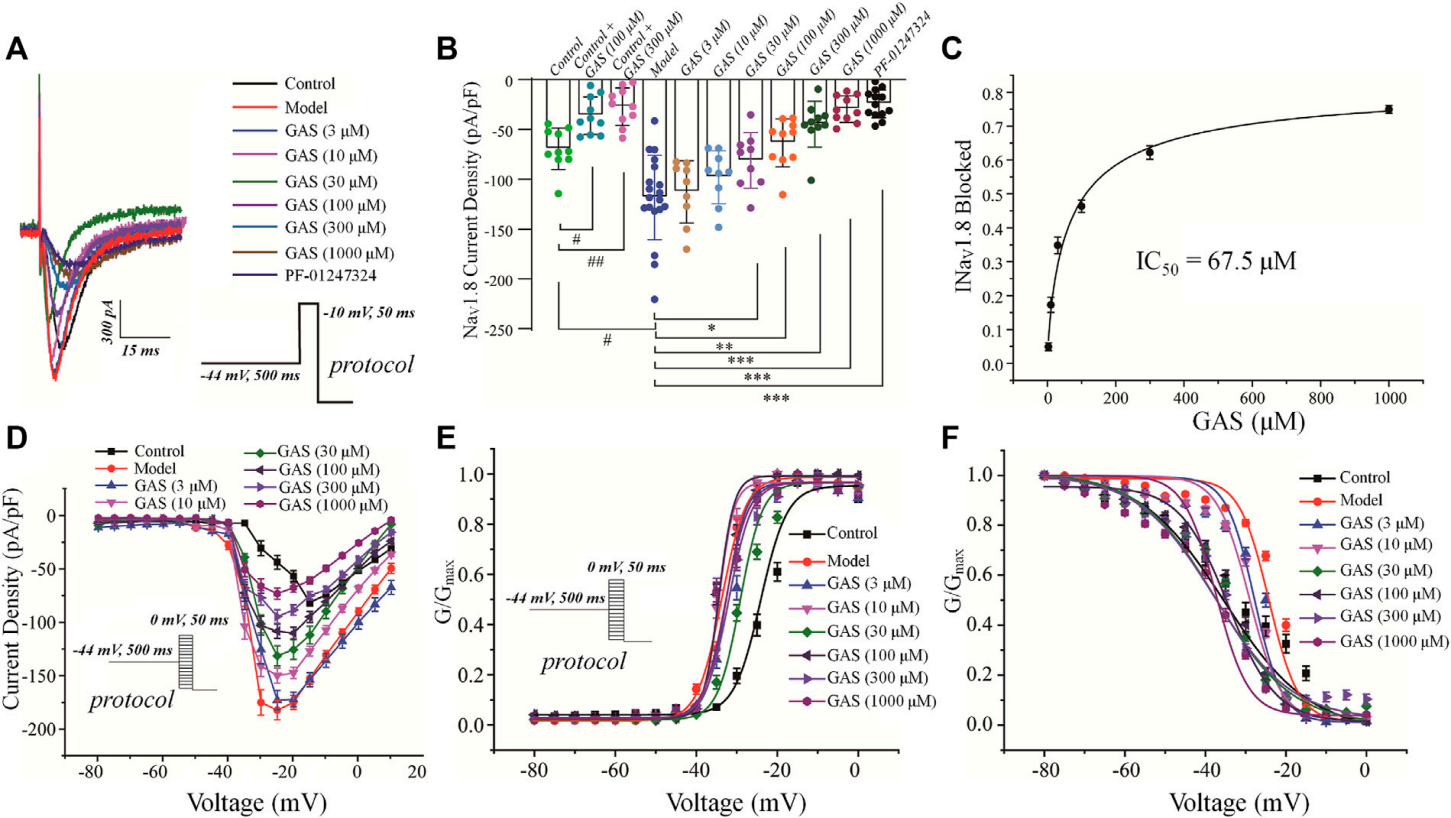
Effects of GAS on Na_V_1.8 channel current in DRG neurons from model rats: **(A)** typical curve of GAS inhibiting Na_V_1.8 channel current of DRG neurons; **(B)** histogram of the inhibitory effects of GAS on the current density; **(C)** concentration-response curves of GAS on Na_V_1.8 current (IC_50_ = 67.5 ± 20.7 μM); **(D)** influence of different doses of GAS on the current density-voltage relationship of DRG neurons. **(E)** effects of different concentrations of GAS on the activation curve of Na_V_1.8 channel; **(F)** influence of different concentrations of GAS on the inactivation curve of Na_V_1.8 channel of DRG neurons (#*p* < 0.05, compared to the control group; *p < 0.05, **p < 0.01, ***p < 0.001, compared to the model group; ANVOA- Bonferroni Test).

In order to isolate TTX-resistant Na_V_1.8 current, TTX (500 nM) was used to block TTX-sensitive component of sodium current and a conditioning prepulse of −44 mV (500 ms) was used to inactivate TTX-resistant Na_V_1.9 channel (see *Materials and Methods* for details). GAS presented a concentration-dependent inhibition of the current density of Na_V_1.8 in DRG neurons in model rats (Figure 4A). Before GAS perfusion, the current density of the control group and the vincristine group were recorded: −69.52 ± 6.5 pA/pF (control, *n* = 10), −118.5 ± 9.8 pA/pF (vincristine, *n* = 19) (Figures 4A,B). Then, the bath solution containing different concentrations of GAS processed the vincristine group, and the current density was recorded was follows: 3 μM GAS, −112.7 ± 9.4 pA/pF (*n* = 10);10 μM GAS, −98.1 ± 8.11 pA/pF (*n* = 10); 30 μM GAS, −77.2 ± 2.2 pA/pF (*n* = 10); 100 μM GAS, −63.5 ± 7.3 pA/pF (*n* = 10); 300 μM GAS, −44.8 ± 6.9 pA/pF (*n* = 10); 1,000 μM GAS, −29.7 ± 3.9 pA/pF (*n* = 10); PF-01247324 (Cat. No.: HY-101383, MCE) (1 μM) −24.5 ± 3.9 pA/pF (*n* = 13) (Figures 4A,B).

Based on the Figure 4B, the inhibitory effects of GAS on Na_V_1.8 sodium channel current from normal DRG neurons depended on relative high concentrations, but GAS could exhibit significant inhibitory effects on Na_V_1.8 sodium channel current from model DRG neurons at the low concentration (30 μM). In addition, when the DRG model neurons were treated with 300 or 1,000 μM GAS, the Na_V_1.8 selective inhibitor PF-01247324 wouldn’t have further significant inhibitory effects on the channel current, indicating that GAS inhibited most of Na_V_1.8 channel current at such concentrations. Certainly, Figure 4B also showed that Na_V_1.8 channel current could be isolated from DRG neuron using such specific biophysical protocol. Besides, the IC_50_ values of inhibitory activities of GAS on the Na_V_1.8 channel current of the model neuron was calculated, i.e., IC_50_ = 67.5 ± 20.7 µM (Figure 4C). The relationship between conductance (G_Nav_1.8) and voltage was presented by scatter plot, which was further fitted with Boltzmann equation to generate activation and inactivation curves.

Besides, GAS had no significant effects on the activation curve of Na_V_1.8 channel, making the inactivation curve shift to the hyperpolarization direction, and the specific results were as follows: The inactivation voltage (V_1/2_) value of the vincristine model group was −24.2 ± 1.2 mV, and the inactivation V_1/2_ of GAS at different concentrations were summarized in Table 1. By further comparing the effects of GAS on the inactivation V_1/2_ of the vincristine model (3 μM GAS group, ΔV_1/2_ = −3.6 mV; 10 μM GAS group, ΔV_1/2_ = −4.9 mV; 30 μM GAS group, ΔV_1/2_ = −13.2 mV; 100 μM GAS group, ΔV_1/2_ = −10.2 mV; 300 μM GAS group, ΔV_1/2_ = −13.5 mV; 1,000 μM GAS group, ΔV_1/2_ = −13.1 mV), it is found that GAS affected the inactivation V_1/2_ of the model in a dose-dependent manner (Figure 4F).

Compared with the neuronal activation curve of the vincristine group, GAS did not affect the activation curve (Figures 4E,E), but all the groups treated with GAS could make the steady-state inactivation curve of conductance (G_Nav1.8_)-voltage shift to hyperpolarization, indicating that GAS only affected the steady-state inactivation curve and hardly influenced the slope of the steady-state inactivation curve of the Na_V_1.8 channel (Figure 4F). The V_1/2_ value inactivating G_Nav1.8_ shifted to the hyperpolarization direction by a dose-dependent manner (Table 2). In short, GAS significantly reduced the current amplitude of the Na_V_1.8 current, mainly mediating the inactivation state of the Na_V_1.8 channel. In summary, GAS significantly decreased the current density of the Na_V_1.8 channel and promoted the inactivation state of the Na_V_1.8 channel, further influencing the dynamics of the Na_V_1.8 channel. Based on the above results (Figures 2F, 4B), GAS significantly suppressed the current density of the Na_V_1.8 channel, which is the main reason for the effective reduction of the peak action potential. In addition, GAS made Na_V_1.8 channels prone to inactivation (namely, more sodium channels were in an inactive state at the relatively negative membrane potential), which in turn produced inhibitory activity.

Additionally, it was also reported that GAS could also inhibit the activities of Na_V_1.6, indicating that GAS had certain broad-spectrum inhibition activity on sodium ion channels (Shao et al., 2017). Furthermore, GAS could decrease the transient sodium current (*I*_NaT_) and increase slowly inactivating potassium currents (*I*_AS_) of streptozotocin-induced painful diabetic neuropathy rat model, exerting analgesic effects. Meanwhile, previous studies also pointed out that GAS had certain inhibitory activities on other ion channels, such as acid-sensing ion channels, which had certain therapeutic significance for pain caused by extracellular acidification or spinal synaptic potentiation (Xiao et al., 2016).

#### The Down-Regulation of GAS on Na_V_1.7 Channel and Na_V_1.8 Channel Protein in DRG Neurons

The up-regulation of Na_V_1.7 and 1.8 has been found in a variety of pain models, such as experimentally induced diabetes and inflammation models leading to mechanical allodynia and thermal pain (Renganathan et al., 2001; Kretschmer et al., 2002; Black et al., 2004; Shields et al., 2012). Additionally, the intrathecal injection of Na_V_1.8 antisense oligonucleotides could partially reduce mechanical allodynia and thermal hyperalgesia by down-regulating the expression of SNS transcripts. Therefore, it was necessary to explore whether GAS had influences on the expression of Na_V_1.7 and Na_V_1.8 channels in DRG neurons of CINP rat model, which was helpful to reveal the inhibitory mechanism of GAS on neurons.

**FIGURE 5:**
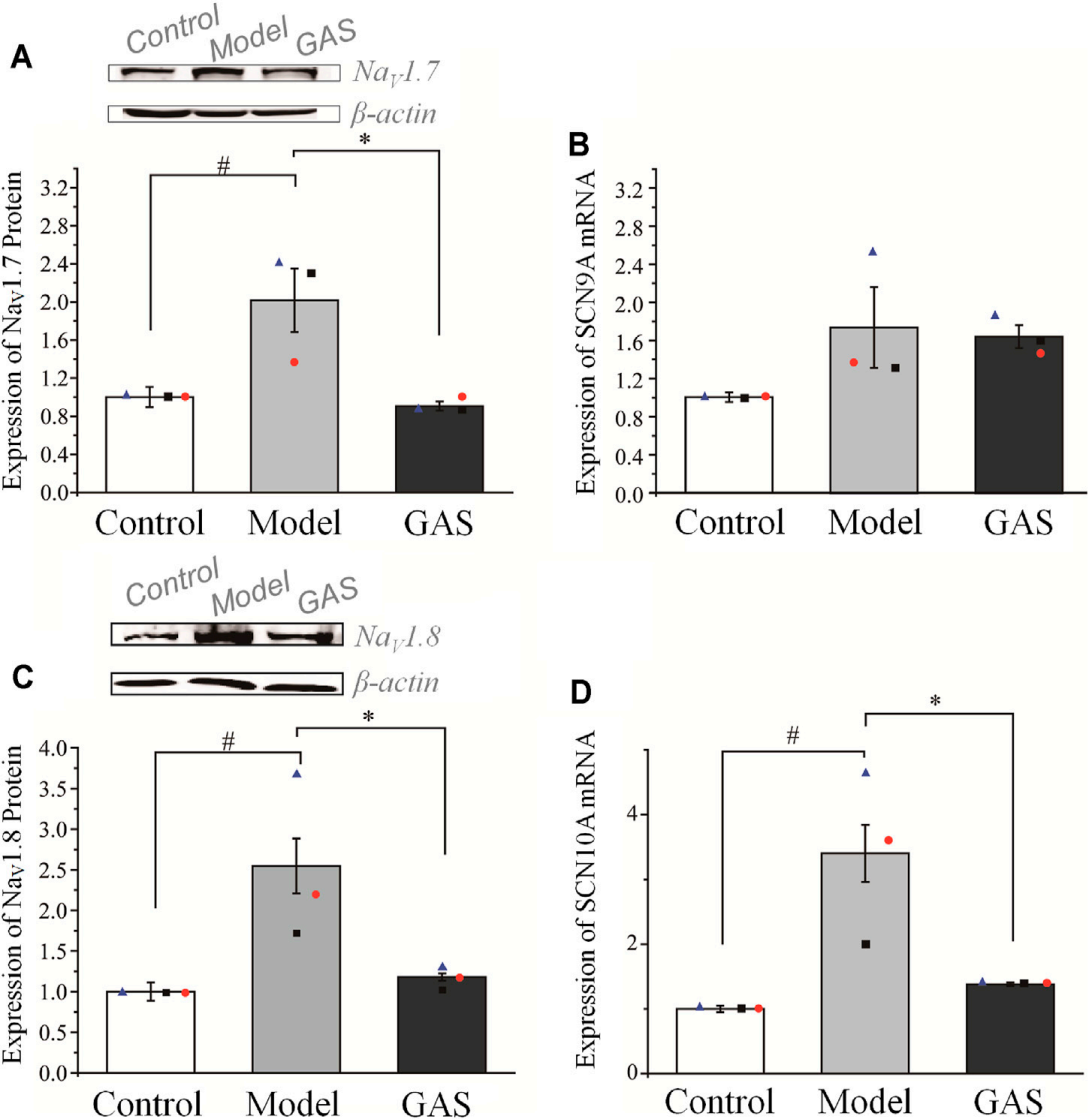
Effects of GAS on the expression level of SCN9A and SCN10A genes and corresponding proteins: **(A)** down-regulation of Na_V_1.7 channel proteins in DRG neurons of vincristine model by GAS (*n* = 3); **(B)** effects of GAS on the expression of SCN9A mRNA in DRG neurons in vincristine model; **(C)** the effects of GAS on the expression of Na_V_1.8 channel protein in DRG neurons (*n* = 3); **(D)** influences of GAS on the expression of SCN10A mRNA in DRG neurons of vincristine model (#*p* < 0.05, compared to the control group; *p < 0.05, compared to the model group; ANVOA- Bonferroni Test).

After the behavioral test on the 14th day, the rats were deeply anesthetized with 3% sodium pentobarbital (50 mg/kg, i.p.), and the DRG neurons were extracted for Q-PCR and WB. GAS had no significant effects on the expression of SCN9A’s mRNA, but significantly inhibited the expression of SCN10A’s mRNA. (SCN9A: 1.74 ± 0.42 in the vincristine group, *n* = 3; 1.64 ± 0.12 in GAS group, *n* = 3, *p* > 0.05; SCN10A: 3.47 ± 0.38 in the vincristine group, *n* = 3; 1.38 ± 0.01 in GAS group, *n* = 3, *p < 0.05), as shown in Figures 5B,D. Next, it was observed that GAS could reduce the upregulation of Na_V_1.7 and Na_V_1.8 channel proteins induced by vincristine (*p < 0.05, *n* = 3), as shown in Figures 5A,C. The above results show that GAS plays an important inhibitory role in resisting the up-regulation of SCN10A mRNA, Na_V_1.7, and Na_V_1.8 channel proteins caused by vincristine. Next, it was observed that GAS could reduce the upregulation of Na_V_1.7 and Na_V_1.8 channel proteins induced by vincristine (*p < 0.05, *n* = 3) (Figures 5A,C). These results showed that GAS played an important role in inhibiting the up-regulation of mRNA of SCN10A, Na_V_1.7 and Na_V_1.8 channel proteins induced by vincristine.

This part verified that Na_V_1.7 and Na_V_1.8 were indeed accumulated in the DRG neurons of the CINP rat model, which was the main cause of pain. Regardless of the level of mRNA or protein expression, GAS has played a vital role in inhibiting the abnormally high expression of Na_V_1.7 and Na_V_1.8. Therefore, GAS not only affects the function of Na_V_1.7 and Na_V_1.8 channels, but also inhibits the protein expression of Na_V_1.7 and Na_V_1.8 channels, indicating that GAS has more abundant regulatory effects than the specific blockers of Na_V_1.7 and Na_V_1.8 channels.

### GAS Counteracts the Up-Regulation of Na_V_1.7&1.8 Protein Distribution Induced by Vincristine

**FIGURE 6:**
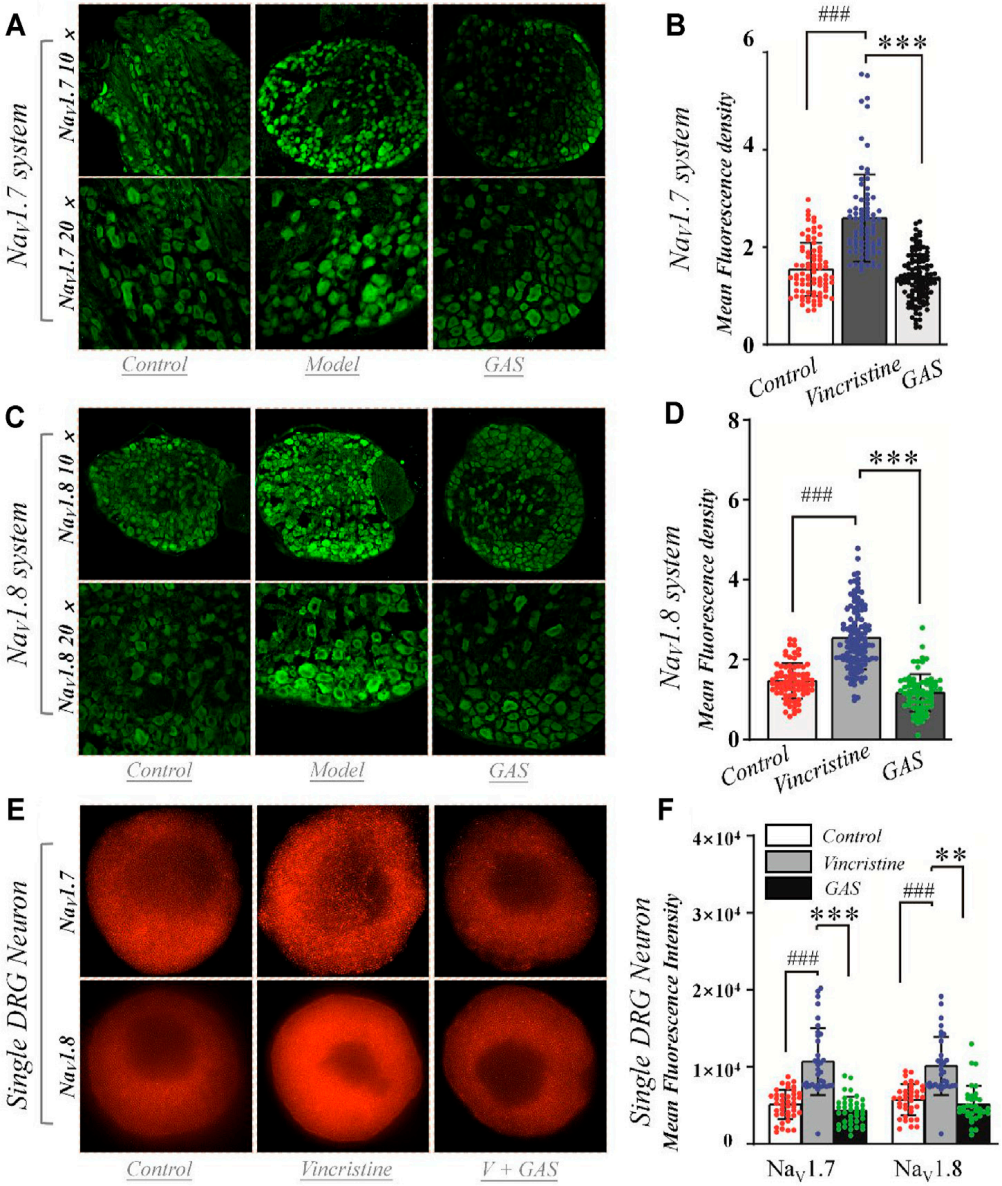
Effects of GAS on the expression of Na_V_1.7 and Na_V_1.8 channel proteins in DRG neurons: **(A)** typical diagram of the effects of GAS on Na_V_1.7 protein expression in DRG ganglion; **(B)** the effects of GAS on the average fluorescence density of Na_V_1.7 protein in DRG tissue (control: *n* = 82, vincristine: *n* = 80, GAS: *n* = 115, N = 4, 5, 5); **(C)** typical diagram of the effects of GAS on Na_V_1.8 protein expression in DRG ganglion; **(D)** the effects of GAS on the average fluorescence density of Na_V_1.8 protein in DRG tissue (control: *n* = 79, vincristine: *n* = 120, GAS: *n* = 74, N = 4, 5, 4); **(E)** SIM image of the distribution of Na_V_1.7 and Na_V_1.8 protein and the depiction of intensity; **(F)** histogram of the influence of GAS on fluorescence intensity of Na_V_1.7 protein (control: *n* = 40, vincristine: *n* = 33, GAS: *n* = 37) and Na_V_1.8 (control: *n* = 37, vincristine: *n* = 32, GAS: *n* = 35) (###*p* < 0.001, compared to the control group; **p < 0.01, ***p < 0.001 compared to the model group; ANVOA- Bonferroni Test).

In order to determine whether GAS affects protein expression, we first observed the effects of GAS on the fluorescence intensity of Na_V_1.7 & 1.8 channels on DRG small-diameter neurons in the L5 segment of model rats from the slice level. And the average fluorescence intensity of each group was as follows: 1) Na_V_1.7 system: control group, 1.5 ± 0.5 (*n* = 82, N = 4); vincristine group, 2.6 ± 0.9 (*n* = 80, N = 5); GAS, 1.4 ± 0.5 (*n* = 115, N = 5) (Figures 6A,B). 2) Na_V_1.8 system: control group, 1.5 ± 0.4 (*n* = 79, N = 4); vincristine group: 2.5 ± 0.8 (*n* = 120, N = 5); GAS group, 1.2 ± 0.5 (*n* = 74, N = 4) (Figures 6C,D), where “n” representing the number of cells and “N” representing the number of ganglions. The above results indicated that GAS could significantly reduce the fluorescence intensity of Na_V_1.7 & 1.8 protein in DRG neurons of model rats.

DRG cells were incubated with vincristine and GAS mixture for 24 h and imaged using structured illumination microscopy (SIM) and confocal fluorescence microscopy. Immunohistofluorescence utilized moiré fringes generated by high-frequency stripe illumination to separate the high-frequency and low-frequency signals of the sample, making the ultrafine structure of the cell be seen. Fluorescence staining results showed that vincristine could up-regulate the distribution of Na_V_1.7 and Na_V_1.8 channel proteins in the DRG neurons, as shown in Figures 6E,F. GAS mixture incubation significantly reduced the enhanced fluorescence intensity of Na_V_1.7 and Na_V_1.8 membrane proteins induced by vincristine (**p < 0.01, Figures 6E,F). Therefore, GAS significantly reduced the fluorescence intensity of Na_V_1.7 and Na_V_1.8 membrane proteins enhanced by vincristine. The results further verified that GAS inhibits the expression of Na_V_1.7 and Na_V_1.8 channels. Nowadays, studies also pointed out that GAS could alleviate the peripheral neuropathy to some extend by inhibiting some signal pathways or receptors, such as the inhibitory activity of GAS on the P38/MAPK signal pathway (Sun et al., 2016; Qin et al., 2021), indicating the various action mechanisms of GAS, which was worthy of further study.

#### Prediction of the Possible Docking Sites of GAS on Sodium Ion Channels

In this part, we constructed the three-dimensional structures of Na_V_1.7 & 1.8 using homology modeling method, and evaluated and optimized the construction results with Ramachandran plots and molecular dynamics (MD) simulations.

##### Na_V_1.7 System

The amino acid sequence of Na_V_1.7 used in homology modeling was consistent with that of the stable-state expression of Na_V_1.7 in HEK293B cell line in this experiment. The SWISS-MODEL server (https://swissmodel.expasy.org/) was used to perform the homology modelling of the target sequence, and the structure of human voltage-gated sodium channel Na_V_1.7 (PDB ID: 6j8g) (Shen et al., 2019) was used for the template based on the results of “Search For Templates.” Then, the constructed 3D structure was subject to 5 ns molecular dynamic simulation to optimize the unreasonable interatomic contact or collision and minimize the conformational energies. Afterwards, the optimized conformation was used for active pockets prediction, and the corresponding molecular docking and MD simulations were performed.

**FIGURE 7:**
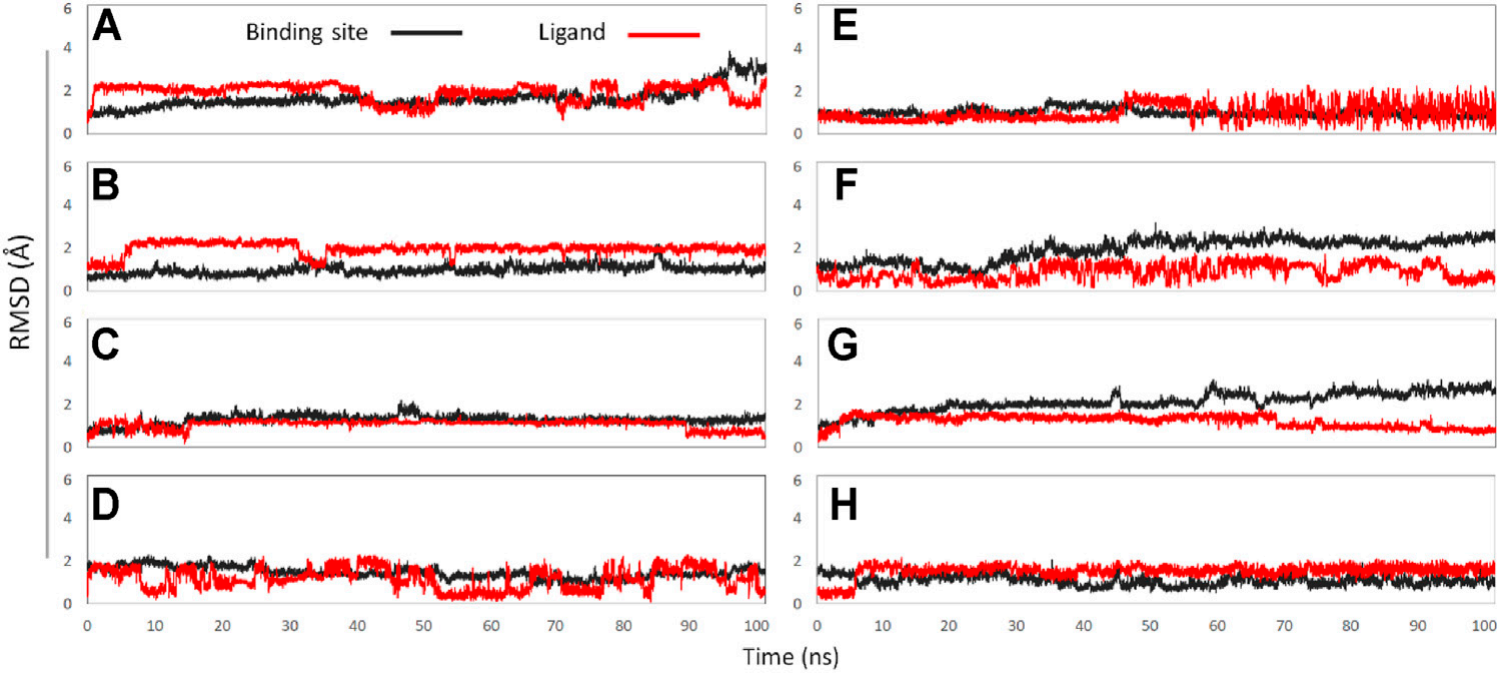
The RMSD values of binding sites (black line) and ligand (red line): **(A)** GAS in Site 1; **(B)** GAS in Site 2; **(C)** GAS in Site 3; **(D)** GAS in Site 4; **(E)** GAS in Site 5; **(F)** GAS in Site 6; **(G)** GAS in Site 7; **(H)** GAS in Site 8.

In this experiment, a total of 8 active binding sites were predicted in Na_V_1.7 (Supplementary Figure S3), and receptor-ligand (Na_V_1.7-GAS) complexes systems were established based on CDOCKER docking method, which were further subjected to 100 ns MD simulation. In addition, RMSD calculation (Figure 7) and binding free energy analysis (MMPBSA.py) were carried out for the simulation trajectories (Table 3). According to Figure 7, it could be found that GAS had varying degrees of volatility at different binding sites, among which the binding on the Site 1 was relatively stable and the absolute binding free energy was relatively large, indicating that Site 1 may be the action site of GAS on Na_V_1.7.

**TABLE 3:** Calculated ΔG_MM/GBSA_ values of GAS in Na_V_1.7 and 1.8.

Constructed systems	Calculated binding free energies (kcal mol^−1^)	
	Na_V_1.7	Na_V_1.8
GAS-Site 1	−28.24 ± 0.24	−9.36 ± 0.17
GAS-Site 2	−8.16 ± 0.27	−16.59 ± 0.26
GAS-Site 3	−19.30 ± 0.21	−17.30 ± 0.30
GAS-Site 4	−7.64 ± 0.25	−4.36 ± 0.25
GAS-Site 5	−9.18 ± 0.08	−12.31 ± 0.45
GAS-Site 6	−14.31 ± 0.27	−11.63 ± 0.25
GAS-Site 7	−20.73 ± 0.29	
GAS-Site 8	−10.01 ± 0.10	

##### NaV1.8 System

Based on literature research and Protein Data Bank (PDB) (https://www.rcsb.org/) search, the crystal structure of Na_V_1.8 has not yet been resolved. Considering that the DRG neurons used in the electrophysiology and Western blotting experiments in this study were derived from SD rats (belonging to Rattus Norvegicus species), NP_058943.2 (Scn10a, Gene ID = 29571, organism = *Rattus norvegicus*) (Yu et al., 2014) was used as the amino acid sequence of Na_V_1.8 for homology modelling. Subsequently, in accordance with the operation of the Na_V_1.7 system, the built 3D conformation was first optimized by MD simulation, and then used for active pocket prediction, molecular docking, and MD simulation (He et al., 2020; Li et al., 2021).

**FIGURE 8:**
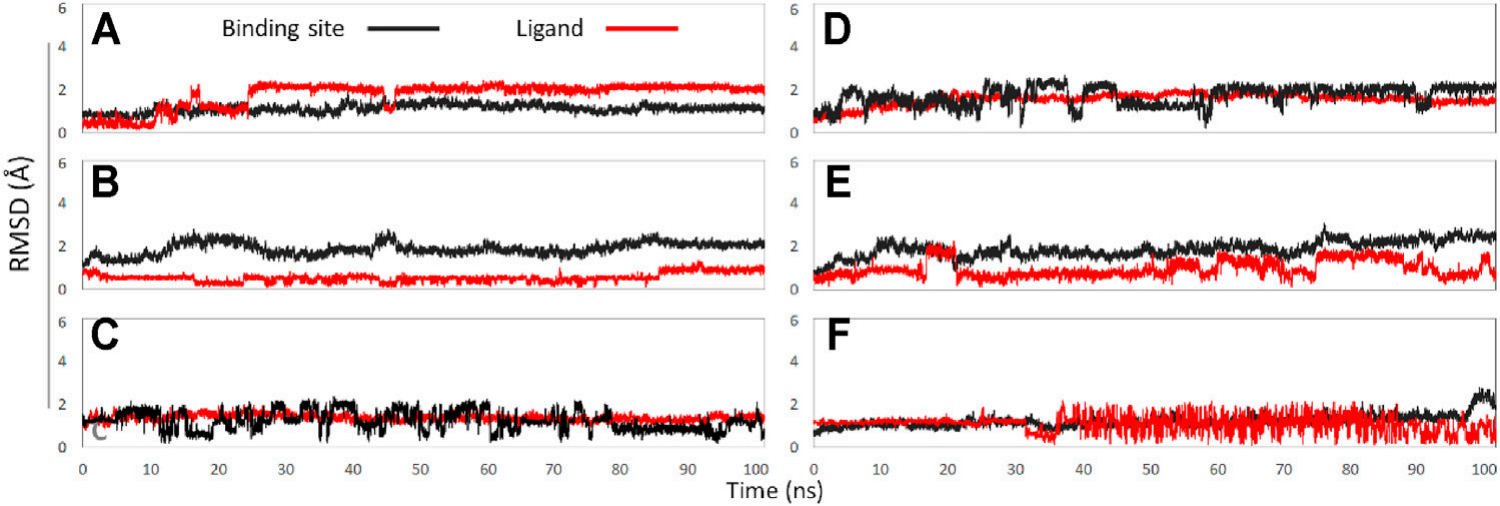
The RMSD values of binding sites (black line) and ligand (red line): **(A)** GAS in Site 1; **(B)** GAS in Site 2; **(C)** GAS in Site 3; **(D)** GAS in Site 4; **(E)** GAS in Site 5; **(F)** GAS in Site 6.

**FIGURE 9:**
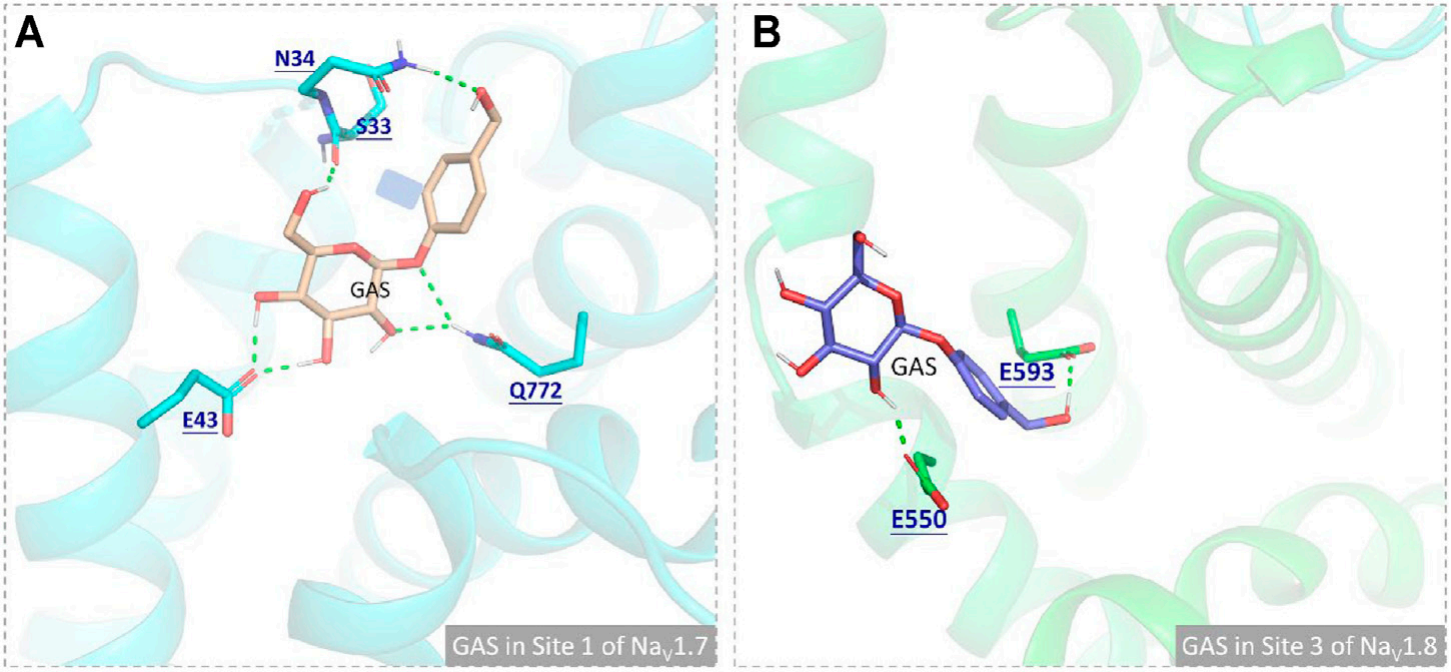
Comparison of the binding interactions of GAS on the active pockets of Na_V_1.7 and Na_V_1.8, and the hydrogen bonds were shown in green dashed lines: **(A)** GAS in Site 1 of Na_V_1.7; **(B)** GAS in Site 3 of Na_V_1.8.

In this part, 6 active binding pockets were predicted in Na_V_1.8 (Supplementary Figure S4), and the corresponding receptor-ligand (Na_V_1.8-GAS) complexes systems were constructed, which were also subjected to 100 ns MD simulation to evaluate the binding interactions between Na_V_1.8 and GAS. According to Figure 8 and Table 3, it could be found that there were varying degrees of difference in the binding interactions of GAS on Na_V_1.8 (from Site 1 to Site 6). By overall comparison of the effects of GAS on Na_V_1.7 and Na_V_1.8, the affinity of GAS on Na_V_1.7 was better than that of Na_V_1.8, indicating that GAS was more inclined to bind to Na_V_1.7, which was basically consistent with the inhibition of GAS on the current density of Na_V_1.7 & 1.8. In addition, we analyzed the most likely binding conformations of GAS on Na_V_1.7 & 1.8, and found that GAS could form 6 hydrogen bonds with residues (E43, N34, S33, and Q772) on the Site 1 of Na_V_1.7 (Figure 9A). However, only 2 residues in Site 3 of Na_V_1.8 could form hydrogen bonds with GAS (Figure 9B), indicating that GAS could bind more stably on Na_V_1.7.

## Conclusion

GAS could modulate the Na_V_1.7 and Na_V_1.8 channels to promote their inactivation. As Na_V_1.7 and Na_V_1.8 channels act on the initial and rising stages of action potentials, GAS could inhibit the abnormal discharge of action potentials of DRG neurons, especially ectopic discharge induced by vincristine. In addition, GAS could significantly down-regulate the total protein expression of Na_V_1.7 & 1.8 proteins. By regulating the channel function of Na_V_1.7 and Na_V_1.8 and the regulation of protein levels, the pain behavior of CINP rats induced by vincristine can be alleviated. In conclusion, GAS not only modulated the biological functions of Na_V_1.7/Na_V_1.8 channels but also down-regulated the protein expression, which expanded the understanding of the action mechanism of GAS as neuromodulator, pointing out the direction for application of natural products in peripheral nerve pain caused by chemotherapy.

## Data Availability

The original contributions presented in the study are included in the article/Supplementary Material, further inquiries can be directed to the corresponding authors.
