# Supplementary material for: Mechanisms Underlying Gastrodin Alleviating Vincristine-Induced Peripheral Neuropathic Pain
Source: Front Pharmacol. 2021 Dec 16;12:744663. doi: 10.3389/fphar.2021.744663 (PMC8716817; doi:10.3389/fphar.2021.744663)
Supplement: Supplementary file 1 [file DataSheet1.PDF]

Supplementary Materials for:

Mechanisms underlying Gastrodin Alleviating Vincristine-induced Peripheral Neuropathic Pain

Xiangyu Wang, **Boxuan Zhang**, **Xuedong Li**, **Xingang Liu**, **Songsong Wang**, **Yuan Xie**, **Jialing Pi**, **Zhiyuan Yang**, **Jincan Li**, **Qingzhong Jia*** and **Yang Zhang***

School of Pharmacy, Hebei Medical University, Shijiazhuang 050017, China.

Corresponding Authors:

*Qingzhong Jia (qizhjia@hebmu.edu.cn), Department of Pharmacology, Hebei Medical University, Shijiazhuang 050017, China;

*Yang Zhang (20162901007@cqu.edu.cn), Department of Medicinal Chemistry, Hebei Medical University, Shijiazhuang 050017, China.

Supplementary materials for:

RESULTS and DISCUSSION

In addition, in order to better illustrate the mechanisms underlying GAS alleviating vincristine induced peripheral neuropathic pain, the influences of GAS on the mechanical and thermal thresholds and the excitability of DRG neurons in normal SD rats were also observed.

(1) The effects of GAS on mechanical and thermal thresholds in the normal rats.

Normal SD rats were intraperitoneally injected with 60 mg/kg GAS for three consecutive days, and mechanical threshold and thermal withdrawal latency were measured on the first and third days respectively, preliminarily judging the influences of GAS on mechanical and thermal injury stimulus thresholds of normal rats. The detailed information were as follows: **DAY 0** (without administration): control group, mechanical threshold, 7.0 ± 1.6 g, thermal withdrawal latency, 19.3 ± 2.3 s; GAS group, mechanical threshold, 6.8 ± 1.4 g, thermal withdrawal latency, 19.4 ± 3.3 s. **DAY 1**: control group, mechanical threshold, 6.7 ± 1.1 g, thermal withdrawal latency, 19.0 ± 3.2 s; GAS group, mechanical threshold, 6.8 ± 2.6 g, thermal withdrawal latency, 18.5 ± 4.3 s. **DAY 3**: control group, mechanical threshold, 7.0 ± 1.4 g, thermal withdrawal latency, 21.5 ± 4.3 s; GAS group, mechanical threshold, 8.1 ± 2.2 g, thermal withdrawal latency, 23.9 ± 3.9 s (**Figure S1**). Obviously, mechanical and thermal threshold didn't increase significantly after three days of GAS administration, indicating that the mechanism of GAS reversing vincristine-induced hyperalgesia was different from that of anesthetic effects.

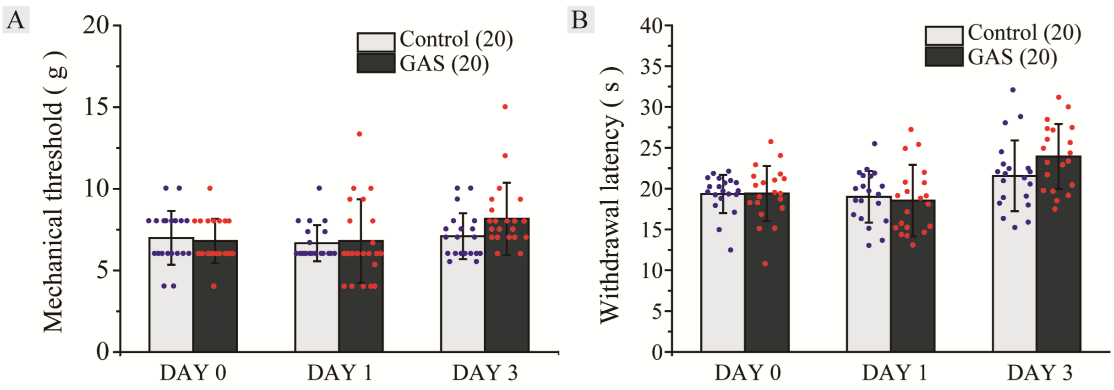

Figure S1. The effects of GAS on alleviating the symptoms of normal SD rats: (A) The

effects of GAS on mechanical paw withdrawal duration of normal SD rats (0 Day, 1 Day, 3 Day); (B) Effects of GAS on plantar thermal pain in normal SD rats, and the thresholds were also measured at 0 Day, 1 Day, 3 Day.

(2) The effects of GAS on the excitability of DRG neurons in normal SD rats.

The DRG neurons of normal SD rats were quickly separated, and then perfused (10 min) with bath solution containing different concentrations of GAS or 500 nM PF-05089771, and the excitability of DRG neurons (number of action potential bursts, rheobase intensity, amplitude, and threshold) was recorded:

<u>Number of action potential bursts</u>: control group = 4.2 ± 1.1 (n = 20), 30 μM GAS = 3.0 ± 1.1 (n = 14), 100 μM GAS = 2.3 ± 0.9 (n = 11), PF-05089771 = 0.9 ± 0.2 (n = 10, ##p < 0.01). Obviously, unlike PF-05089771, GAS couldn't significantly reduce the number of action potential bursts in normal SD rats.

<u>Rheobase</u>: control group = 369.6 ± 44.9 pA (n = 30), 30 μM GAS = 490.0 ± 55.1 pA (n = 20), 100 μM GAS = 582.3 ± 16.9 pA (n = 17, ##p < 0.01), PF-05089771= 740.0 ± 78.8 pA (n = 10, ###p < 0.001). Thus, higher concentration of GAS (100 μM) showed similar effects to that of PF-05089771, significantly increasing the rheobase of the action potential.

amplitude control group = 118.3 ± 4.1 mV (n = 27), 30μM GAS = 111.0 ± 7.1 mV (n = 19) 100 μM GAS = 105.4 ± 6.6 mV (n = 17), PF-05089771 = 89.6 ± 10.1 mV (n = 10. P < 0.01). Therefore, different from PF-05089771, GAS could not affect the amplitude of action potential of normal neurons.

<u>threshold</u>: group = -25.6 ± 0.9 mV (n = 25), 30 μM GAS = -23.1 ± 1.3 mV (n = 14), 100 μM GAS = -22.1 ± 1.3 mV (n = 18, #p < 0.05), PF-05089771= -17.5 ± 1.9 mV (n = 10, ###p < 0.001). GAS of 100 μM and 500nM PF-05089771 could both significantly increase the threshold of action potentials.

Based on the effects of GAS on the excitability of DRG neurons in normal SD rats, it could be found that GAS could increase the threshold and rheobase of action potentials to a certain extent, but had fewer effects on the number and amplitude of action potential, indicating that DRG neurons from normal SD rats were less sensitive to GAS compared to that of model DRG neurons.

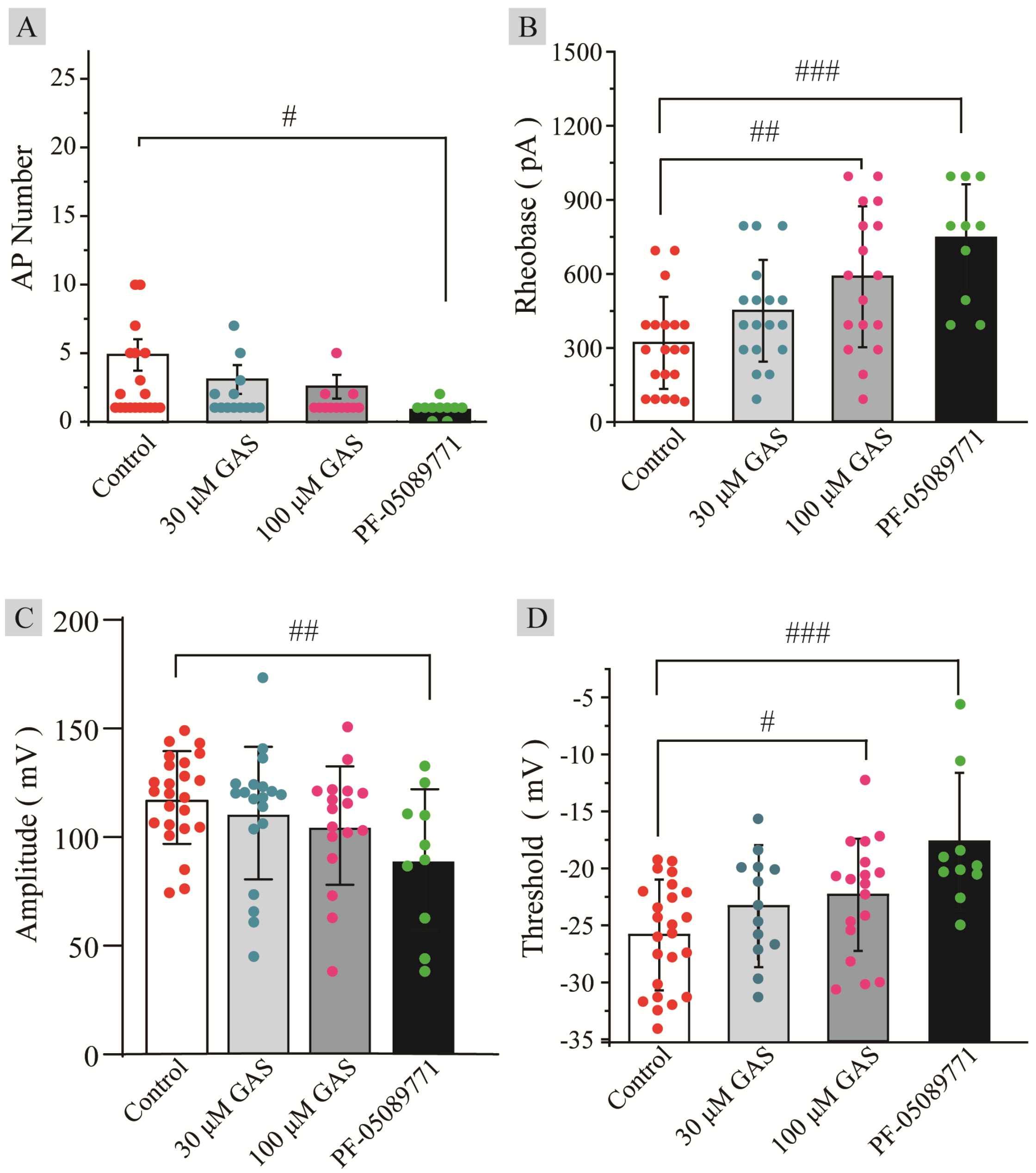

Figure S2. The influences of GAS on the excitability of small-sized DRG neurons from normal SD rats: (A) the effect of GAS on the number of action potentials fired by DRG neurons in each group; (B) histogram of the effects of GAS on action potential rheobase of DRG neurons in each group; (C) the influence of GAS on the amplitude of the first peak of action potential fired by DRG neurons; (D) regulation of GAS on the firing threshold of action potentials of DRG neurons in each group (#p < 0.05, ##p < 0.01, ###p < 0.001, compared to the control group, ANVOA- Bonferroni Test).

(3) Prediction of the active binding sites in $Na_V1.7$ and $Na_V1.8$.

In this experiment, a total of 8 active binding sites were predicted in $Na_V1.7$ and 6 active binding pockets were predicted in $Na_V1.8$ (**Figure S3 and Figure S4**).

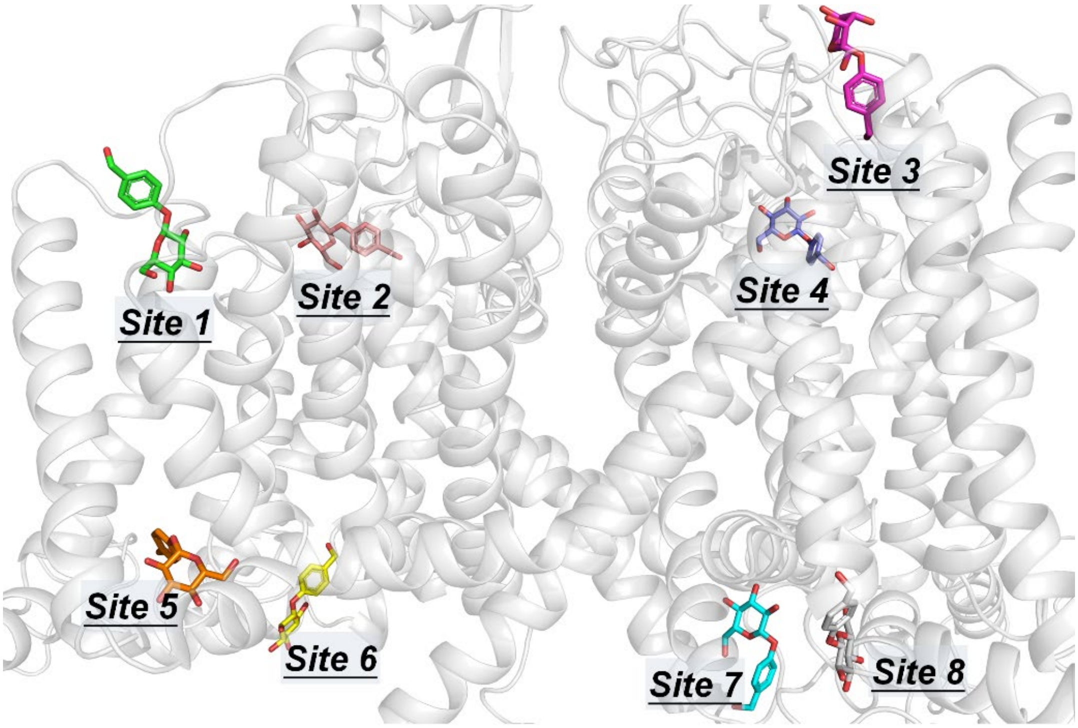

Figure S3. The predicted binding sites of GAS on $Na_V1.7$.

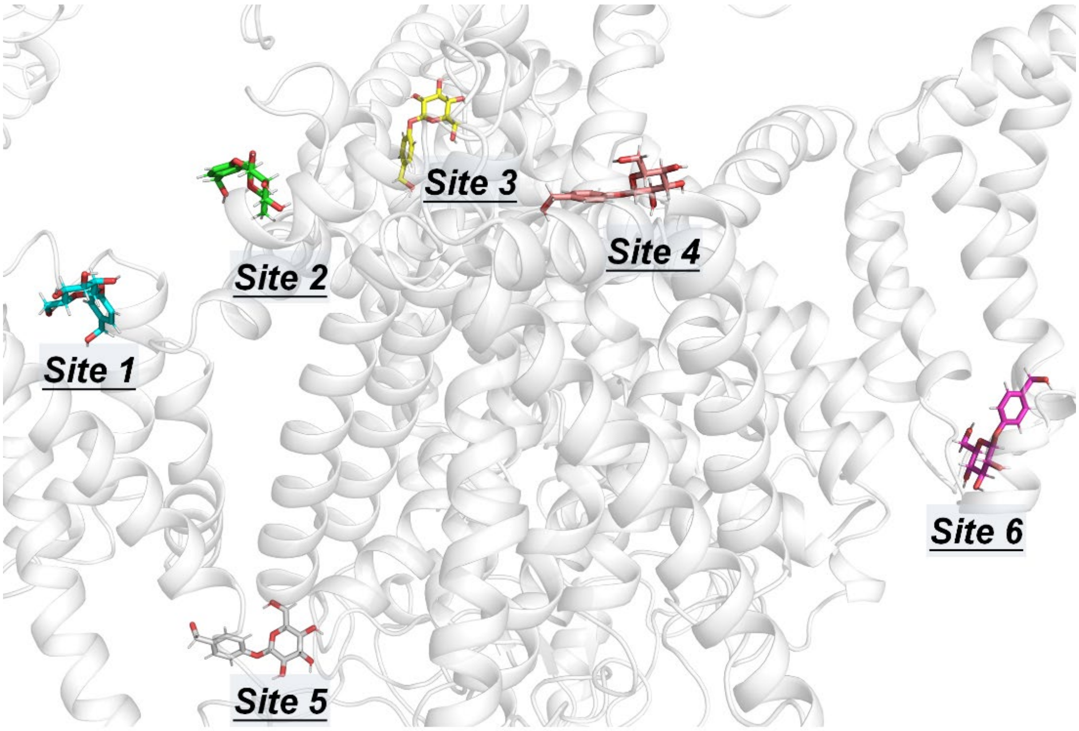

Figure S4. The predicted binding sites of GAS on $Na_V1.8$
